# Maturation of Oral Microbiota in Children with or without Dental Caries

**DOI:** 10.1371/journal.pone.0128534

**Published:** 2015-05-28

**Authors:** Pernilla Lif Holgerson, Carina Öhman, Agneta Rönnlund, Ingegerd Johansson

**Affiliations:** 1 Department of Odontology/section of Pedodontics, Umeå University, Umeå, Sweden; 2 Department of Odontology/section of Cariology, Umeå University, Umeå, Sweden; LSU Health Sciences Center School of Dentistry, UNITED STATES

## Abstract

**Background:**

The aim of this longitudinal study was to evaluate the oral microbiota in children from age 3 months to 3 years, and to determine the association of the presence of caries at 3 years of age.

**Methods and findings:**

Oral biofilms and saliva were sampled from children at 3 months (n = 207) and 3 years (n = 155) of age, and dental caries was scored at 3 years of age. Oral microbiota was assessed by culturing of total lactobacilli and mutans streptococci, PCR detection of *Streptococcus mutans* and *Streptococcus sobrinus*, 454 pyrosequencing and HOMIM (Human Oral Microbe Identification Microarray) microarray detection of more then 300 species/ phylotypes. Species richness and taxa diversity significantly increased from 3 months to 3 years. Three bacterial genera, present in all the 3-month-old infants, persisted at 3 years of age, whereas three other genera had disappeared by this age. A large number of new taxa were also observed in the 3-year-olds. The microbiota at 3 months of age, except for lactobacilli, was unrelated to caries development at a later age. In contrast, several taxa in the oral biofilms of the 3-year-olds were linked with the presence or absence of caries. The main species/phylotypes associated with caries in 3-year-olds belonged to the *Actinobaculum*, *Atopobium*, *Aggregatibacter*, and *Streptococcus* genera, whereas those influencing the absence of caries belonged to the *Actinomyces*, *Bergeyella*, *Campylobacter*, *Granulicatella*, *Kingella*, *Leptotrichia*, and *Streptococcus* genera.

**Conclusions:**

Thus, during the first years of life, species richness and taxa diversity in the mouth increase significantly. Besides the more prevalent colonization of lactobacilli, the composition of the overall microbiota at 3 months of age was unrelated to caries development at a later age. Several taxa within the oral biofilms of the 3-year-olds could be linked to the presence or absence of caries.

## Introduction

The microbiota of the oral cavity and other parts of the gastro-intestinal (GI) tract develops from virtual sterility at birth into one of the most heavily colonized parts of the human body [1,2,3,4], but with distinct bacterial communities at the various anatomical niches [5]. In the mouth alone, more than 700 taxa have been identified, with approximately two thirds belonging to cultivable species (named or unnamed), and one-third belonging to the uncultivable phylotypes (www.homd.org) [6].

Microbial variations during the first few years of life lead to the establishment of a stable bacterial ecosystem in the GI tract, including the mouth [4]. Generally, facultative anaerobic genera, such as *Streptococci* and *Actinomyces*, are the initial colonizers, succeeded by more anaerobic genera, such as *Bifidobacteria* in the gut, and *Veillonellae* and *Fusobacteria* in the mouth [1, 7]. Molecular based methods such as PCR, cloning and Sanger sequencing, microarrays, and multiplex pyro- or Illumina sequencing, have provided insight into both cultivable and uncultivable bacterial species in health and disease. For example, several studies suggest that changes in microbial flora during the early variation period, and in the final composition of the gut flora, are related to negative health outcomes, such as allergy, obesity, and intestinal diseases during childhood [4, 8, 9], and obesity and myocardial infarction in adults [10, 11]. There are few studies examining the impact of early microbial colonization in the mouth, but early acquisitions of the cariogenic species, *Streptococcus mutans* and *Streptococcus sanguinis*, have been associated with increased and decreased risks of dental caries, respectively [12, 13].

Microarray and multiplex sequencing are less labor intense when compared to single species PCR and sequencing, after cloning, for in-depth characterization of the microbiota. Although microarrays provide taxonomic resolution at the species level or of a group of similar species of predetermined bacteria, multiplex sequencing determines the whole array of bacteria. The limitation of the latter method relates to sequencing errors and the length of the obtained sequence, which affects the taxonomic resolution. These limitations have been met by quality filtering, and the obtained sequence length has increased over time to approximately 450 bp with the 454 FLX+ pyrosequencing methods. The most commonly used gene in multiplex sequencing, and for the taxonomic assignment of oral microbiota, is 16S rRNA, as in the HOMD (Human Oral Microbiome Database) curated database on common oral bacteria (www.HOMD.org) [14].

Dental caries is a highly prevalent polymicrobial infectious disease characterized by demineralization of tooth tissues, and dysbiosis of the tooth colonizing microbiota [15, 16, 17, 18]. Several studies have applied newer multiplex sequencing methods such as 454 FLX pyrosequencing; in order to identify caries associated microbiota [19, 20, 21]. However, the results obtained from these studies, conducted mostly in young children with early childhood caries (ECC), lack uniformity. The microbial species reported to be associated with ECC are, *Streptococcus*, *Veillonella*, *Lactobacillus* (especially in dentin caries), *Olsenella*, *Actinomyces*, *Prevotella*, *Granulicatella*, *Leptotrichia*, *Propionibacterium*, *Megasphaera*, and *Scardovia* [18, 22]. In contrast, several studies have found species such as *Capnocytophaga*, *Fusobacteria*, *Tannerella*, *Phorpyromonas*, *Abiotrophia*, and *Streptococcus*, on healthy tooth surfaces [21, 22].

The aim of the present study was to compare the oral microbiota at 3 months and 3 years of age, and to assess the relationship between these microorganisms and the presence or absence of caries, at 3 years of age. Both 454 FLX+ pyrosequencing complemented by HOMIM microarray (Human Oral Microbe Identification Microarray), and species specific PCR, for taxonomic identification at various levels, were performed in this study.

## Material and Methods

### Ethics statements

The study was approved by the Regional Ethical Review Board in Umeå, Umeå University (http://www.epn.se), Sweden (Dnr 07-100M), and was conducted according to the principles described in the Declaration of Helsinki. Written informed consent was obtained from all caregivers.

### Subjects and study design

**Fig 1:**
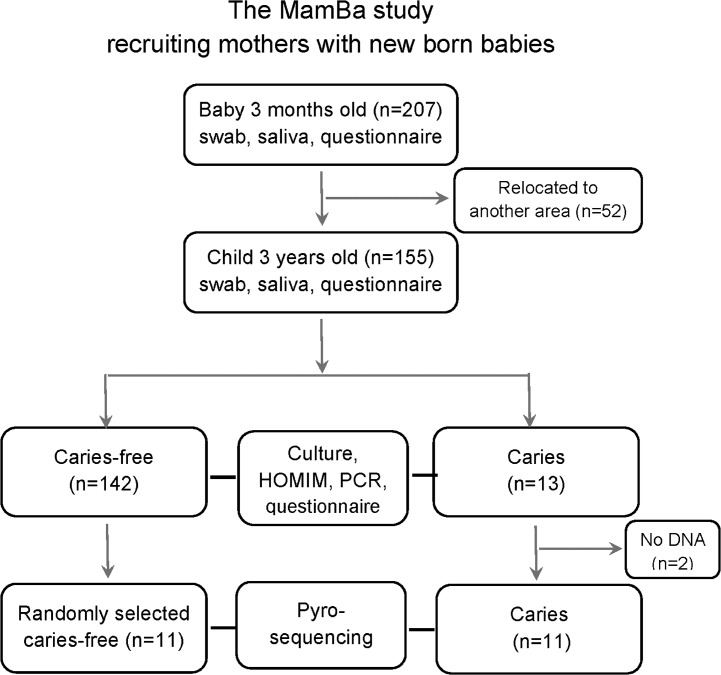
Flow chart diagram. Description of number of study participants and dropouts, data collection and analyses and caries status at 3 years of age.

This is a longitudinal cohort study (acronym, MamBa, Mamma-Barn, Swedish for Mother-Child) of infants from the age of 3 months to 3 years. Information on the association between mode of delivery and feeding in relation to microarray-assessed microbiota has been described previously [23, 24]. Briefly, all mothers with babies born between September 2007 and January 2009 in a small inland town or a coastal university city in Northern Sweden, and with available contact details, were eligible for the study. However, only those parents, who were likely to stay in the area, were invited to provide consent for their child’s participation in the study (Fig 1). Children with a severe disease, and those associated with complicated pregnancies or deliveries, were excluded. A total of 207 (52%) mothers consented to participate in the study. At 3 months of age, the child visited the hospital along with his/her mother, who was then asked to complete a questionnaire regarding the feeding mode (exclusive or partial breast feeding, or exclusive formula feeding), mode of delivery, gestational age at birth, infant health (allergy, infections, stomach discomfort), use of antibiotics or products containing probiotic bacteria, use of a pacifier, and presence of teeth. Samples were collected from the infant at this stage. At 3 years of age, the child attended a regular oral examination at the nearest Public Dental clinic, with his/her mother/father, as part of the free annual examinations running from 3 to19 years of age, in Sweden. During this visit, an oral examination was performed and samples were collected by a specialist in pediatric dentistry (PLH). The caregiver was asked to complete another questionnaire covering the same topics as that at 3 months of age. Information regarding infant body weight and length, at birth, at 3 months, and at 3 years of age, was obtained from child health care and medical records.

### Caries scoring

Caries was registered using a mirror and probe, under good lighting conditions, and bitewing radiographs were used when approximal surfaces were unavailable for visual inspection. The criteria for caries detection were as described by the World Health Orginization [25]. Non-cavitated lesions (initial caries lesions) were identified when the surfaces presented a “chalky-white” appearance. Decayed (d), missing (m), and filled (f) surfaces (s) were recorded, and the sum (dmfs score), including carious lesions in enamel and dentine, were calculated.

### Samplings at 3 months and 3 years of age

At 3 month of age, the buccal mucosa, tongue, and alveolar ridges were swabbed carefully using sterile cotton swabs (Applimed SA, Chatel-St-Denis, Switzerland). At 3 years of age, this procedure was repeated, and the teeth were scraped with a sterile wooden toothpick, for the presence of dental plaque, which, along with the mucosa-adherent biofilm, was immediately pooled into Eppendorf tubes (Sarstedt, Nümbrecht, Germany) with 200 μl TE-buffer (10 mM Tris, 1 mM EDTA, pH 7.6). In addition, at both 3 months and 3 years of age, approximately 1 mL saliva was collected from the child, into ice-chilled, sterile test tubes, as described previously [23]. All samples were obtained between 1–3 hours (mean 2 hours) after the latest meal, and stored at −80°C.

### Bacteria cultivation

Colony-forming units (CFU) of mutans streptococci per mL of saliva, were estimated by cultivation on mitis salivarius sucrose agar supplemented with 0.2 U bacitracin at 37°C in 5% CO_2_. Lactobacilli CFUs were estimated by culture on Rogosa agar (Merck, Darmstadt, Germany) anaerobically incubated at 37°C for 48–72 h. Total viable counts were cultured on blood agar (Columbia base agar, supplemented with 5% horse blood), and anaerobically incubated at 37°C for 48–72 h. This was done for all children at 3 months and 3 years of age.

### DNA extraction

Bacterial DNA was purified from all swab and swab/tooth samples, using the GenElute Bacterial Genomic DNA kit (Sigma Aldrich, St. Louis, MO), according to the manufacturer’s instructions, and as described previously [23]. The final quality and quantity of the DNA was evaluated using a Nanodrop 1000 spectrophotometer (Thermo Scientific, Wilmington, DE). Polymerase chain reaction (PCR) of *Streptococcus mutans* and *Streptococcus sobrinus* was performed using HotStarTaq Mastermix (Qiagen, Hamburg, Germany) with specific primers described in Yano et al [26]. For *S*. *mutans* the forward primer was 5’AGCCATGCGCAATCAACAGGTT 3’ and the reverse 5’CGCAACGCGAACATCTTGATCAG 3’, and for *S*. *sobrinus* the forward primer was 5’GAAACCAACCCAACTTTAGCTTGGAT 3’ and the reverse 5’ATGGAGTGATTTTCCATCGGTACTTG 3’ with the thermal cycling conditions: 15 minutes at 95°C; 30 cycles of 30 seconds at 95°C, 30 seconds at 60°C, 1 minutes at 72°C; followed by 5 minutes at 72°C [26].

### Pyrosequencing and data processing

Oral samples, from all children with caries at 3 years of age, and an equal number of randomly selected children who were free of clinical signs of caries, were selected for microbiota characterization by 454 FLX+ pyrosequencing. The V3-V4 hypervariable region of the 16S rRNA gene was amplified by PCR, using the universal forward primer, 347F, and the reverse primer, 803R [27]. For sample identification, fusion primers with unique barcode sequences according to the “Guidelines for Amplicon Experimental Design (www.454.com), by Roche Diagnostics, were created. DNA was amplified under the following running conditions: initial denaturation at 94°C for 3 min, followed by 30 cycles at 94°C for 15 s, 58°C for 15 s, and 72°C for 30 s, with a final extension at 72°C for 8 min.

Amplicon processing and 454 sequencing were conducted at the Lund University Sequencing Facility (Faculty of Science, Lund, Sweden), using the Lib-A chemistry supported by the FLX+ platform. Complementary runs were performed on a 454 Roche Junior apparatus at Umeå University (Department of Immunology). After cleaning (Agencourt AMPure XP, Beckman Coulter, Brea, CA) and inspection (DNA 1000 kit on a 2100 Bioanalyzer, Agilent Technologies, Palo Alto, CA), the amplicons were quantified (Quant-iT ds DNA assay kit, Invitrogen, Carlsbad, CA, and a Quantifluor fluorometer, Promega, Madison, WI), and diluted to obtain a total of 1×10^7^ copies/uL. Titration and library production (aiming at 10–15% enrichment) were performed using emulsion PCR, and the Lib-A kit (Bi-directional sequencing, Roche Diagnostics, Branford, CT). DNA positive beads were enriched, counted (Innovatis CASY particle counter, Roche Innovatis, Bielefeld, Germany), processed (XLR70 sequencing kit, Roche Diagnostics), and loaded onto a picotiter plate for pyrosequencing on a 454 Life Sciences Genome Sequencer FLX+ machine (Roche Applied Science, Penzberg, Germany). In Umeå, Roche’s bench-top machine, GC Junior sequencer (Roche Applied Science, Penzberg, Germany), was used according to the manufacturer’s guidelines.

Sequences were processed using the Quantitative Insights into Microbial Ecology (QIIME, version 1.8.0) software package. Sequences with a minimum length of 300 base pairs after primer sequence removal, with correct barcode sequences, maximum 1 incorrect base pair in the primer sequences, and meeting default quality criteria for homopolymers and quality scores in QIIME, were included in the analysis. Furthermore, the reverse primer was removed, and sequences starting with the reverse primer were reverse complemented. The sequences were clustered into Operational Taxonomic Units (OTUs) at a sequence similarity of 97% in the 16S rRNA chimera checked Human Oral Microbiome Database [14], using USEARCH [28]. OTUs with a single sequence were removed, and representative sequences for each of the remaining OTU clusters were taxonomically assigned against the Human Oral Microbiome Database (www.HOMD.org) [14]. Sequences with ≥98.5% identity were taxonomically assigned to the species (named or unnamed species, or uncultivable species) level, while those between 97.0 and <98.5% were assigned to the genus level.

### Microarray analysis

Microarray identification of microbes was done in all children, at 3 months and 3 years of age, using the HOMIM facility at the Forsyth Research Institute, Boston, MA, (http://mim.forsyth.org/homim.html) as described previously [23, 29]. The HOMIM (Human Oral Microbe Identification Microarray) microarray holds 422 oligonucleotide probes, targeting more than 300 bacterial taxa. Hybridization signals are read on a scale of six (0–5) with a lower limit of detection of 10^4^ cells.

### Data analyses

IBM SPSS Statistics (version 22.0; IBM Corporation, Armonk, NY, USA) was used for descriptive analyses and univariate testing of differences and associations. Normally distributed variables are presented as means with 95% confidence interval (CI), and differences between group means were tested using parametric testing (unpaired t-test). Non-normally distributed variables (including all sequence variables) are presented as medians with range, and non-parametric testing (Mann-Whitney U test) were used to test if the subjects in the two age groups and in the caries-affected and caries-free groups, respectively, were samples from the same or different populations. Chi-square test was used to test differences in-group frequency distributions. For taxa comparisons, a p-value of ≤0.008 was considered statistically significant after accounting for multiple testing by the false discovery rate. Univariate comparisons were not applied for single taxa due to the combination of small groups and a high number of repeated tests. For other variables a p-value of <0.05 was considered statistically significant.

Rarefaction curves were calculated to compare microbial richness among the clinical samples, and principal coordinate analysis (PCoA) to compare the phylogenetic diversity (β diversity) of these samples, using QIIME.

Multivariate principal component (PCA) was used to identify clustering of subjects, and partial least square (PLS) (SIMCA P+, version 12.0, Umetrics AB, Umeå, Sweden) to identify taxa associated with caries status [18, 30, 31]. Models with pyrosequencing data only, and those with the addition of lactobacilli and streptococci by culture and PCR, were tested. Variables were autoscaled to unit variance, and cross-validated predictions of Y were calculated. Clustering of subjects is displayed in a score-loading plot, and the importance of each x-variable is displayed in a loading plot. Variables with a 95% confidence interval for the PLS correlation coefficient that did not include zero, were considered statistically significant. The Q^2^ value gives the capacity of the x-variables to predict the outcome (caries).

## Results

At 3 years of age, 155 of the original 207 participating children had an oral examination. The remaining 52 did not visit the clinic mainly due to relocation to another area. The number of erupted teeth, among the 3-year-old children who participated in the study, ranged from 16–20 with a mean of 19.8 teeth (Table 1). Thirteen children had caries, while 142 were caries-free. Mean dmfs (sum of decayed missing filled surfaces) among the 13 children with caries was 1.6 (1.0–4.0), with 38% of the caries lesions located in the enamel only, whereas 62% had extended into the dentin. A majority (90%) of the caries lesions were in the tooth fissures, and 10% on the smooth buccal surfaces, whereas the lingual or approximal surfaces of the teeth remained unaffected. The mean body weight at birth, 3 months and 3 years of age, as well as the proportion of boys versus girls, breastfed versus not, and those delivered by Cesarean section did not differ between children who developed caries and those who did not (Table 1). However, at 3 years of age, children who developed caries were taller than those who did not (Table 1).

**Table 1:** Study group characteristics at birth, 3 months and 3 years of age.

	Basic study cohort				Children with microbiota analyses		
		Caries-free	Caries		Caries-free	Caries	
	All children^a^	(n = 142)	(= 13)	p-value^b^	(n = 11)	(= 11)	p-value^b^
**At birth (n = 207)**							
weight (kg)	3.5 (3.4–3.6)	3.5 (3.4–3.6)	3.6 (3.2–4.0)	0.678	3.6 (3.4–3.9)	3.7 (3.3–3.7)	0.777
length (cm)	50.1 (49.7–50.4)	50.1 (49.7–50.4)	50.9 (49.3–52.4)	0.241	50.2 (48.9–51.5)	51.5 (50.0–53.1)	0.422
preterm delivery (%)^c^	22.3	22.8	15.4	0.534	0.0	18.2	0.138
cesarean delivery (% yes)	19.8	19.6	23.1	0.760	9.1	27.3	0.269
**3 months of age (n = 207)**							
boys / girls (%)	47.8 / 52.2	49.0 / 51.0	30.8 / 69.2	0.203	18.2/81.8	36.4/63.6	0.338
weight (kg)	6.1 (6.0–6.2)	6.1 (6-0-6.3)	6,0 (5.6–6.5)	0.666	6.3 (6.0–6.6)	6.0 (5.5–6.6)	0.252
length (cm)	60.8 (60.4–61.2)	60.7 (60.4–61.1)	61.4 (59.8–62.9)	0.408	60.9 (59.3–62.5)	61.6 (59.9–63.3)	0.802
breastfed (% yes)^d^	88.9	89.2	84.6	0.613	0.0	18.2	0.138
number of teeth (mean, min-max)	0.03 (0–3)	0.02 (0–3)	0.17 (0–2)	0.223	0 (0–1)	0.2 (0–2)	0.306
total bacteria (^10^log CFU /mL saliva)	7.55 (7.49–7.61)	7.57 (7.51–7.63)	7.27 (7.03–7.49)	0.013	7.49 (7.24–7.74)	7.28 (7.01–7.55)	0.131
lactobacilli in saliva							
% yes by culture	27.8	26.0	46.2	0.003	36.4	45.5	0.414
^10^log CFU /mL	1.01 (0.77–1.26)	0.99 (0.73–1.24)	1.4 (0.23–2.57)	0.423	1.57 (0.04–3.10)	1.08 (0–2.39)	0.509
mutans streptococci in saliva (% yes)	0	0	0	NA	0	0	NA
**3 years of age (n = 155)**							
boys / girls (%)	47.7 / 52.3	49.3 / 50.7	30.8 / 69.2	0.201	45.5 / 54.5	36.4 /63.6	0.665
weight (kg)	14.9 (14.5–15.2)	14.9 (14.5–15.2)	14.7 (13.1–16.2)	0.711	14.2 (12.4–16.1)	14.7 (12.9–16.5)	0.741
length (cm)	95.4 (94.7–96.1)	95.1 (94.4–95.9)	98.0 (95.2–100.8)	0.027	93.1 (88.0–98.1)	97.9 (94.3–101.4)	0.077
number of teeth (mean, min-max)	19.8 (16–20)	19.8 (18–20)	19.9 (16–20)	0.393	19.9 (18–20)	20.0 (20–20)	0.420
dmfs (mean, min-max)	0.13 (1.0–4.0)	0	1.6 (1.0–4.0)	NA	0	1.6 (±0.92)	NA
total bacteria (^10^log CFU /mL saliva)	7.44 (7.36–7.52)	7.44 (7.36–7.53)	7.44 (7.11–7.78)	0.994	7.51 (7.32–7.69)	7.36 (6.99–7.74)	0.462
lactobacilli in saliva							
% yes by culture	5.5	3.0	17.3	<0.001	9.1	18.2	0.484
^10^log CFU /mL	0.18 (0.05–0.31)	0.09 (0–0.18)	1.13 (0.03–2.24)	0.062	0.24 (0–0.76)	0.89 (0–1.93)	0.238
mutans streptococci in saliva							
% yes by culture	21.9	18.6	53.8	0.060	18.2	54.6	0.057
^10^log CFU /mL	0.80 (0.52–1.05)	0.62 (0.37–0.89)	2.53 (1.07–4.00)	0.015	0.57 (0–1.68)	2.05 (0.49–3.61)	0.101
*S*. *mutans*							
% yes by PCR	16.2	16.4	61.5	<0.001	9.1	54.5	0.022
*S*. *sobrinus*							
% yes by PCR	0.7	0.7	0.0	0.759	9.1	0.0	0.306
Intake of sweet fruit soup^e^	19.7	16.8	54.5	0.031	27.2	44.4	0.705

Data are presented as means (95% CI) or proportions (%). Differences between groups were tested with Student´s *t*-test or a Chi2 test, respectively.

a) numbers varies by age as displayed

b) p-value <0.05 statistical significant. Tested between caries diseased and caries-free groups

c) gestational week 38 or earlier

d) exclusively breast fed infants

e) intake of sweet fruit soup. Measured with a questionnaire, frequency of intake per week. Proportion presented is based on an intake more than 3 times a week.

DNA from two children with caries, ended during the 3-month analyses with HOMIM and PCR (Fig 1), and these children could therefore not be represented in the pyrosequencing analyses, resulting in a total of 11 children in the caries group and 11 in the caries-free group. However, all 13 children with caries were included in the other analyses performed in this study (culture, PCR, and HOMIM microarray).

### Culture, 16S rRNA Gene Sequencing and Microarray detection

The mean bacterial count estimated from culture on the anaerobically incubated blood agar plates was 3.2x10^7^ with a range from 3.8x10^3^-1.6x10^8^. From the 44 samples analyzed by pyrosequencing, 932,510 sequences passed quality filtering, with a mean number of 20,514 (95% CI 17,744–23,284) sequences per sample, and a variation of 5,119 to 44,625 sequences. The original sequence data is available at http://dx.doi.org/10.6084/m9.figshare.1384833. All 932,510 sequences could be clustered into 376 OTUs at 97% identity against the HOMD database. Eight of these OTUs could not be assigned to the bacterial kingdom, while another 26 presented with a single sequence. The remaining 342 OTUs represented 7 phyla, or divisions (*Actinobacteria*, *Bacteriodetes*, *Firmicutes*, *Fusobacteria*, *Proteobacteria*, *SR1*, *TM7*), and 72 genera (S1 Table). Fifty-eight OTUs had a sequence identity of <98.5%, and could only be assigned to the genus level, while 284 sequences had an identity of ≥98.5%. The latter 284 sequences represented 196 unique HOT numbers in the HOMD database; named (n = 115) or unnamed (n = 43) species, and uncultivable phylotypes (n = 38). The HOMIM microarray gave a positive signal (score ≥1) for 151 unique species/phylotypes.

### Microbiota at 3 months versus 3 years of age

#### Cultivation and PCR

The total number of bacteria (CFU/mL saliva on blood agar) did not differ between samples collected at 3 months and 3 years of age (Table 1). Mutans streptococci were not detected by culture, pyrosequencing, or microarray at 3 months of age, but at 3 years of age 16% had become colonized with *S*. *mutans* and 1 child with *S*. *sobrinus*. *Notably*, girls were more frequently colonized with mutans streptococci (by cultivation) than boys, at 3 years of age (28.8 versus 13.9%, p = 0.029).

#### Pyrosequencing and Microarray

**Fig 2:**
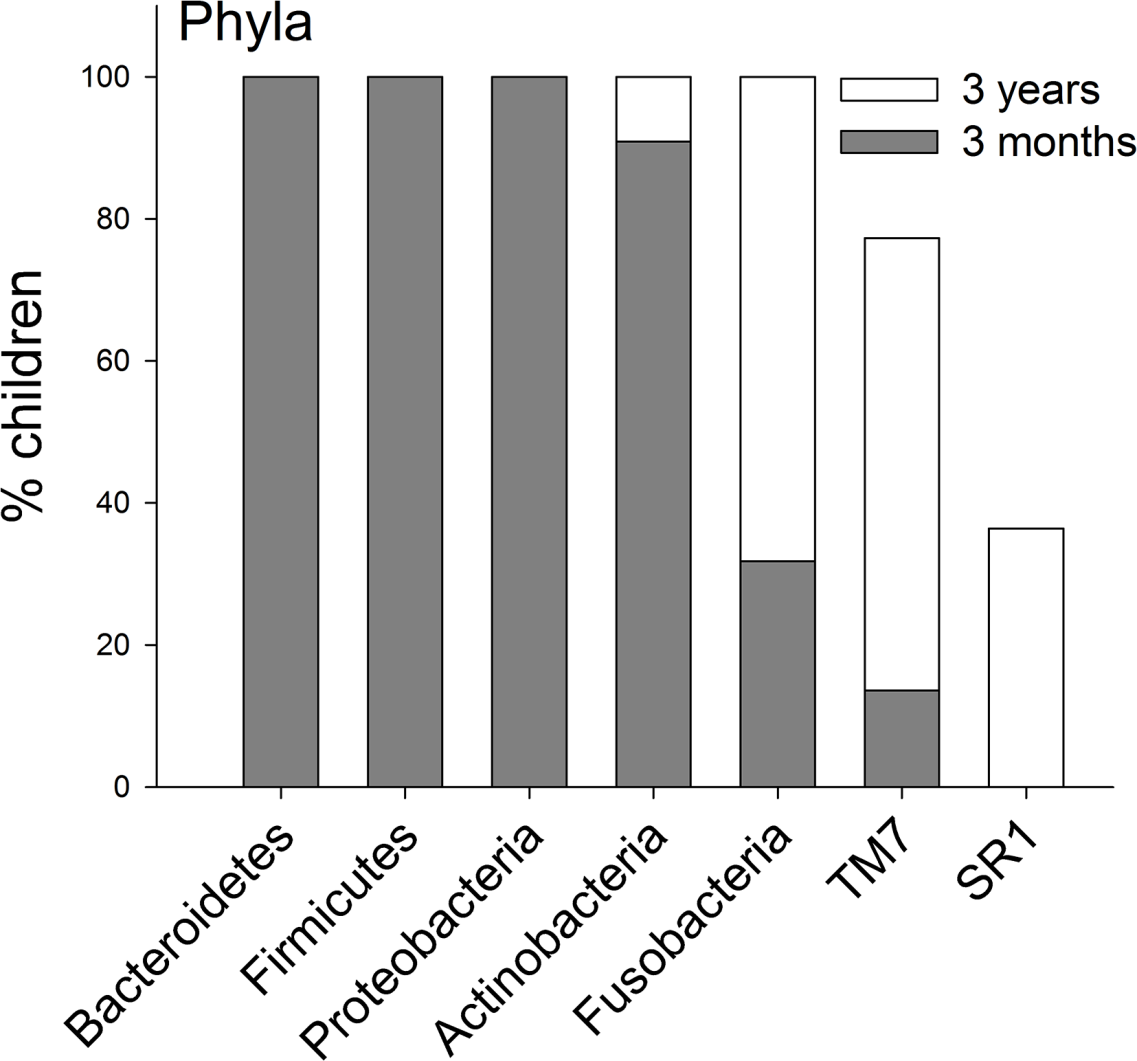
Phyla prevalence. Prevalence at 3-months and 3-years of age of detected phyla.

**Fig 3:**
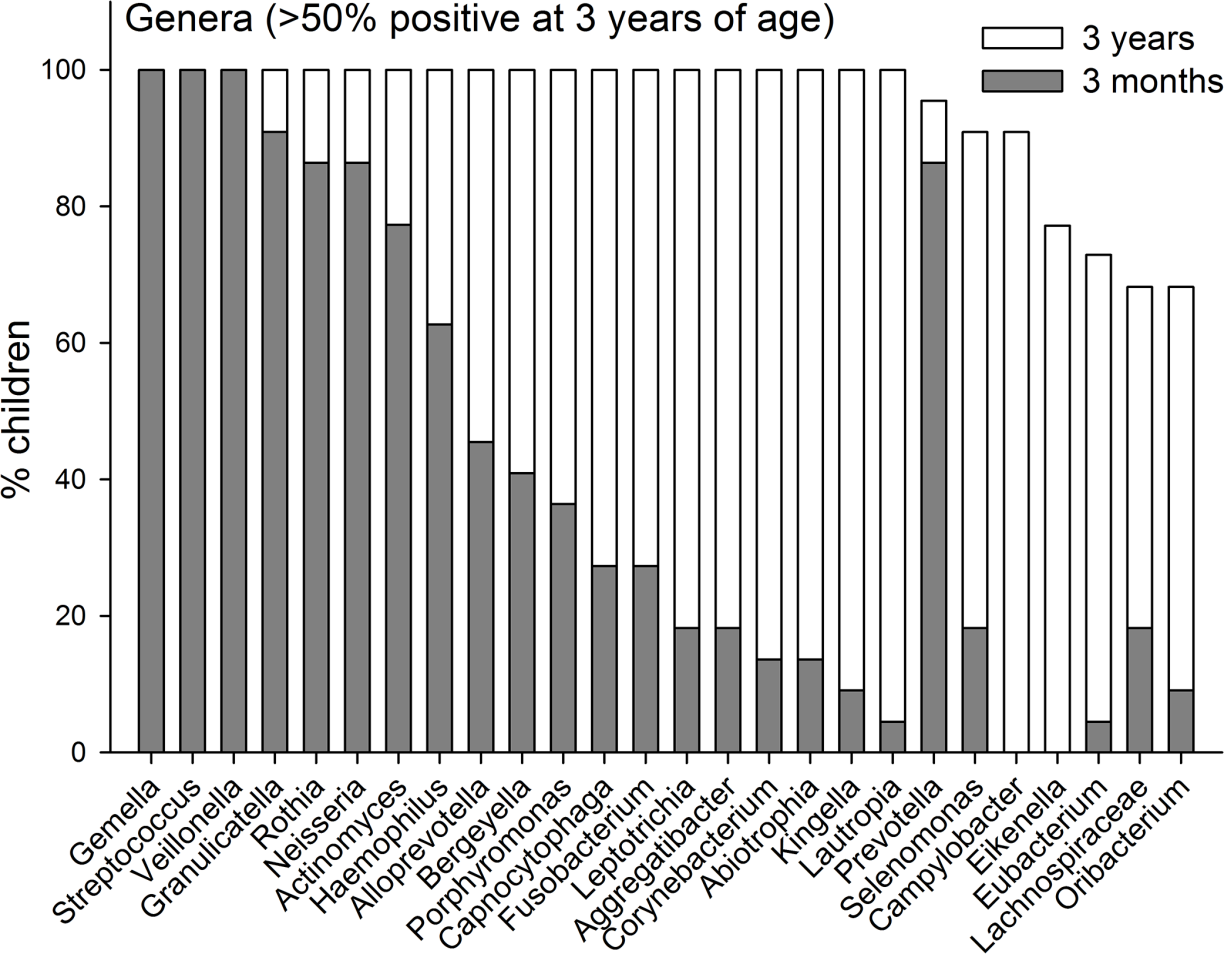
Genera prevalence. Prevalence at 3-months and 3-years of age of genera detected in >50% of the children at 3 years of age.

**Fig 4:**
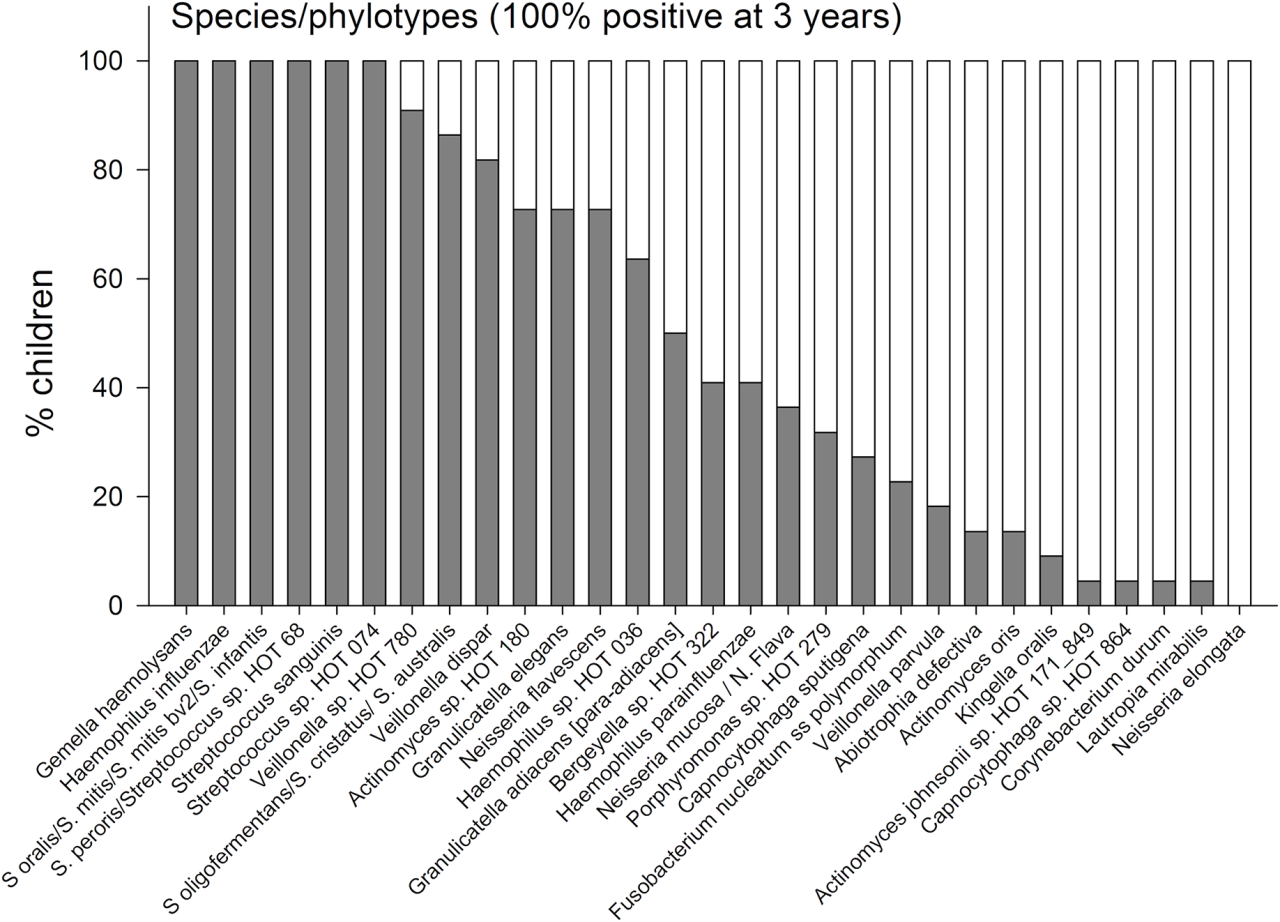
Species/phylotype prevalence. Prevalence at 3-months and 3-years of age of species/phylotypes detected in all (100%) of the children at 3 years of age.

**Fig 5:**
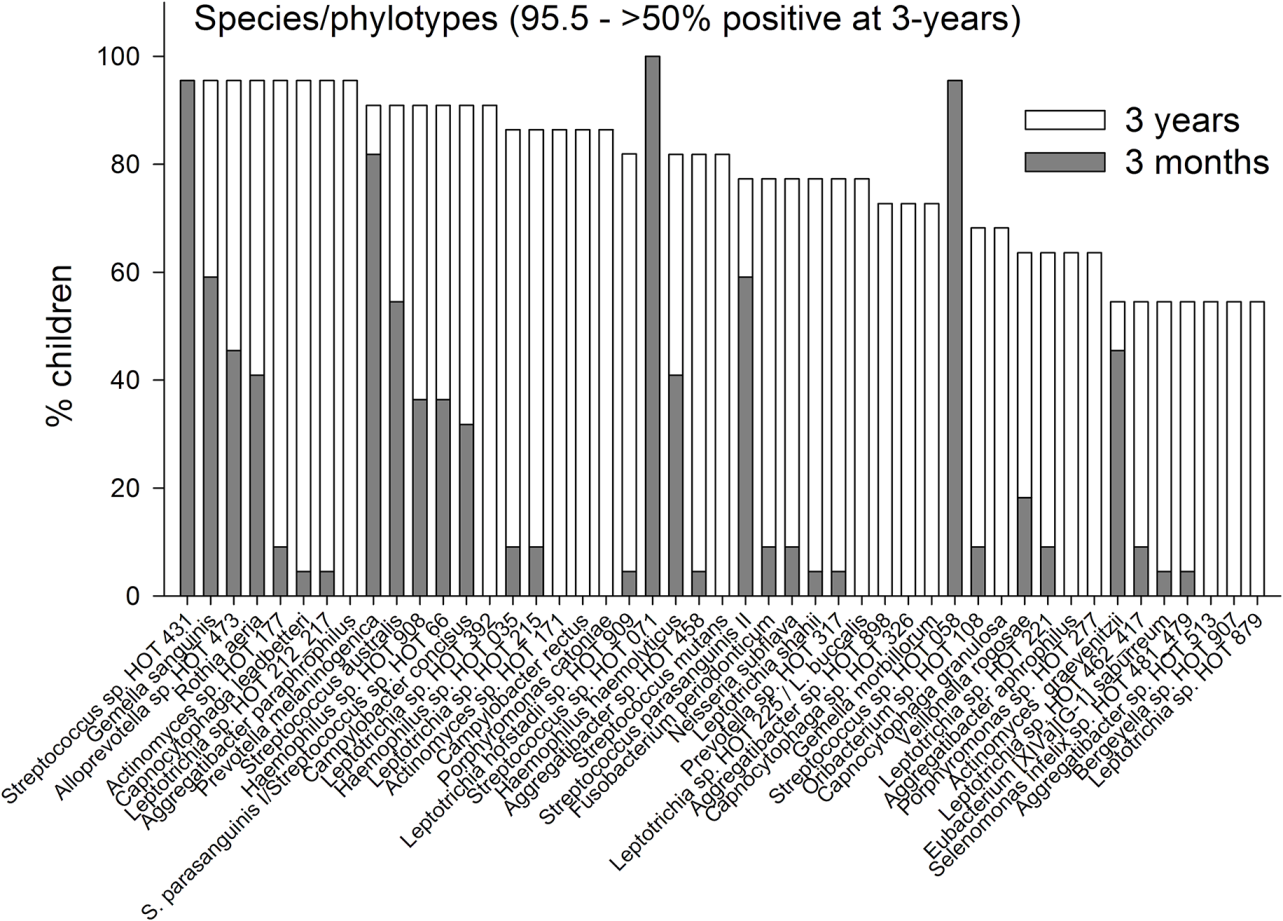
Species/phylotype prevalence. Prevalence at 3-months and 3-years of age of species/phylotypes detected in >50% but <100% of the children at 3 years of age.

The prevalences of the 7 identified phyla are compared for the ages 3-months and 3-years in Fig 2. In Fig 3 the prevalences of genera are present for genera that could be detected in >50% of the 3-year olds. Data for all detected genera are shown in S1 Table. All 196 unique HOT species/phylotypes identified by pyrosequencing were present among the 3-year-old children, but only 176 were observed among the 3 months old infants. The mean (min–max) number of unique species/phylotypes was 31 (14–59) among the 3-month-old infants and 89 (67–128) in the 3-year-old children. In addition, the 3 month and 3-year-old children had 16 (3–28) and 31 (24–42) OTUs, respectively, defined at the genus level. Comparisons of the prevalence of species/phylotypes found in all children at 3-years of age are shown in Fig 4, and for species/phylotypes found in less than 100% but more than 50% are shown in Fig 5. Data for all detected species/phylotypes are shown in S2 Table. A full description of the core microbiome at age 3 months and 3 years are shown in S3 Table.

**Fig 6:**
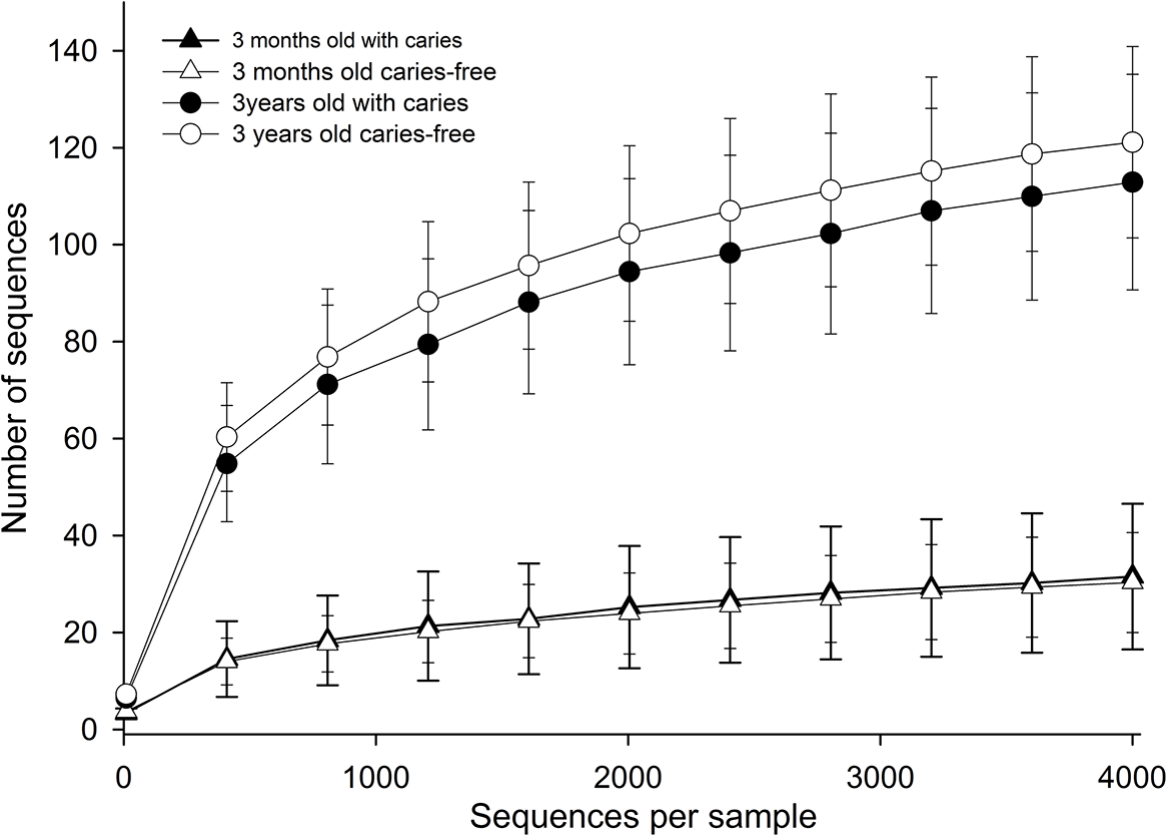
Rarefaction curves. Curves from children at 3 months and 3 years of age, stratified by caries development at 3 years of age. Comparisons include 11 children with caries, and 11 caries-free children.

Significantly lower species richness was confirmed in 3 months compared to 3 year olds in rarefaction analyses (Fig 6). The HOMIM microarray identified 56 unique species/phylotypes among the 3-month-old infants, and 94 unique species/phylotypes among the 3-year-old children.

**Fig 7:**
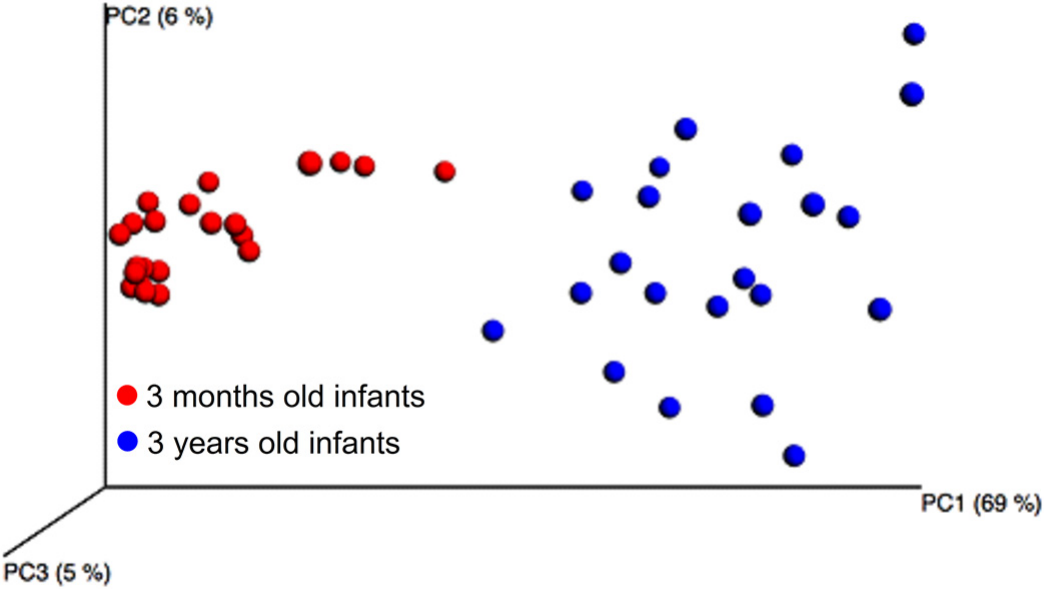
PCoA clustering analysis by age. Red dots indicate samples from the 3-month-old infants, and blue dots indicate samples from the 3-year-olds.

PCoA modeling (weighted and unweighted) of pyrosequencing taxa revealed distinct clustering of the 3-month-old children from the 3-year olds (Fig 7). Similarly, PLS multivariate modeling with age as the dependent variable, and pyrosequencing, or HOMIM identified species/phylotypes as independent variables, revealed 2 significant components with explanatory capacities (R^2^) of 87.6% and 97.2%, and predictive capacities (Q^2^) of 77.5% and 89.8%, respectively (S1 Table). Three genera (*Streptococcus*, *Veillonella*, and *Gemella*) were present in all children at both 3 months, and 3 years of age, whereas another 3 genera (*Escherichia* (*E*. *coli*), *Staphylococcus* (*S*. *epidermidis*), and *Pseudomonas* (no species with >98.5 identity) were significantly more prevalent (p < 0.008) at 3 months compared with 3 years of age. In addition, 2 lactobacillus species (*L*. *crispatus* and *L*. *gasseri)*, and one Streptococcus phylotype (*Streptococcus sp*. *HOT 058*) were more prevalent, a borderline significance, in 3-months old infants than 3-years old children by pyrosequencing (S2 Table). Totally, 23 genera were significantly less prevalent (p < 0.008) at 3 months compared to 3 years of age (S1, S2 and S3 Tables).

### Microbiota in children with or without caries at 3 years of age

#### Cultivation and PCR

Children who had caries at 3 years of age had significantly lower CFU/mL saliva of total bacteria during infancy (3 months) than those who did not (Table 1). The proportion of children with lactobacilli (culture) in saliva was higher both at 3 months and 3 years of age, in the children who had caries at 3 years of age compared to those who did not (Table 1). Further, at 3 years of age, the CFU/mL saliva of total bacteria and the proportion of children colonized with mutans streptococci were higher in children with caries when compared to those who were caries-free (Table 1). Thus, slightly more than half of the children in the former group had mutans streptococci (culture), mainly *Streptococcus mutans* (PCR) compared to less than every 5^th^ child in the latter group (Table 1). Only one child with caries was positive for *Streptococcus sobrinus* by PCR analysis (Table 1).

#### Pyrosequencing and Microarray

**Fig 8:**
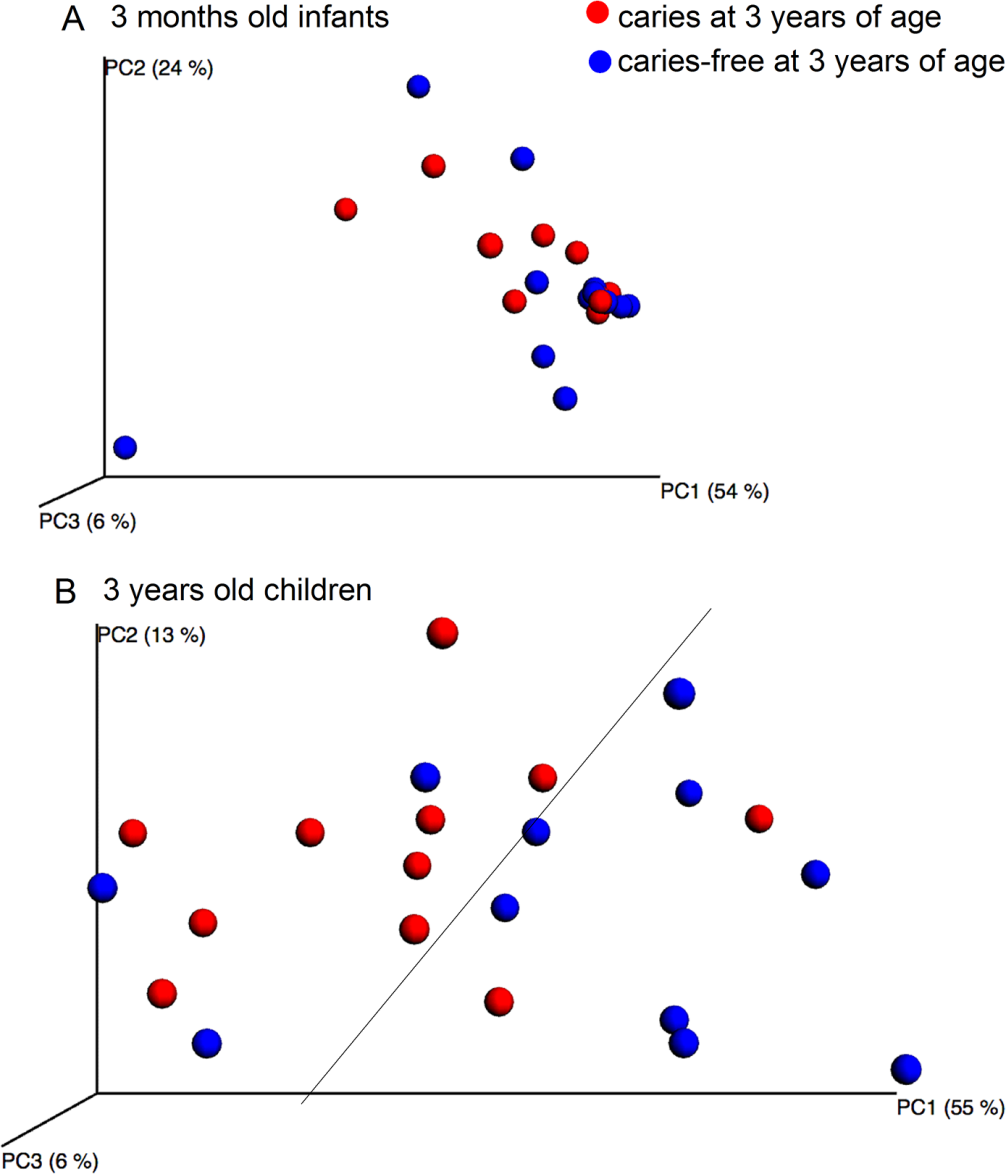
PCoA clustering analysis by caries status. The upper figure (A) shows the microbiota variation between 3-month-old infants who developed caries at 3 years of age (red dots) and those who did not (blue dots). The lower figure (B) shows the microbiota variation between 3-year-old children who had caries (red dots) and those who did not (blue dots).

**Fig 9:**
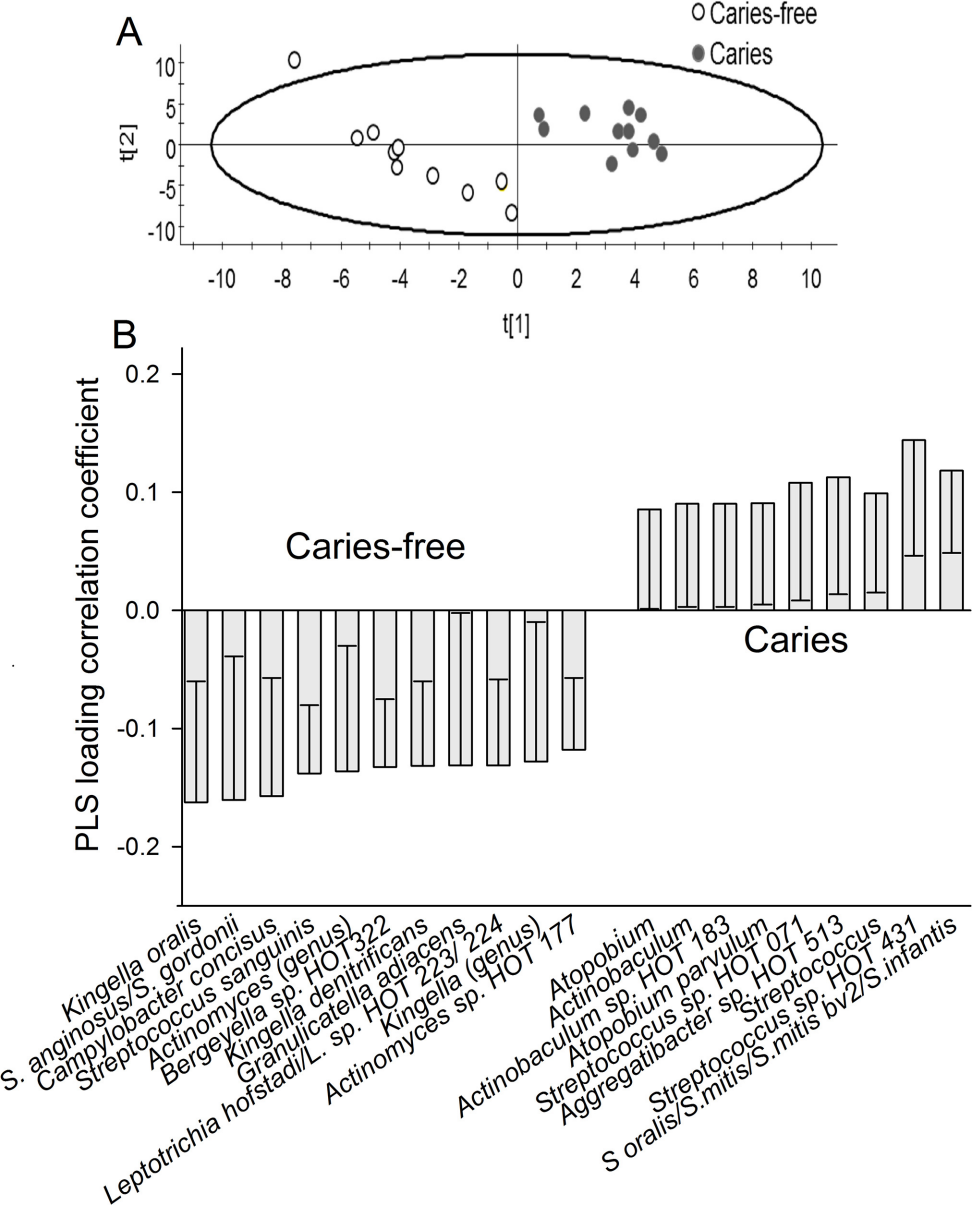
Partial least-squares analysis (PLS) of microbiota associated with having caries or not at 3 years of age (A) Scatter-loading plot illustrating clustering of children having caries or being caries-free. (B) Column loading plot illustrating taxa associated with having caries or not (caries-free). The PLS model employed caries and caries-free allocations as y, and pyrosequencing and HOMIM taxa as the x-block.

Species richness, determined by pyrosequencing of samples collected at 3 months and 3 years of age, did not differ between children with or without caries at the age of 3 months (Fig 7). Further, unweighted or weighted PCoA did not display any cluster formation based on the pyrosequencing determined microbiota at 3 months of age (Fig 8A), and no significant component was found by PLS when pyrosequencing and HOMIM microarray determined microbiota were used as independent variables, and caries status as the dependent variable. However, PCoA tended to separate the 3-year-olds with caries from those without caries (Fig 8B), and two significant components were identified by PLS, when pyrosequencing and HOMIM microarray taxa were used as independent variables, and caries status as the dependent variable. The predictive power of this model was 21.0%. The most influential variables in the 3-year-old caries-free children were (in alphabetical order): *Actinomyces* genus, *Actinomyces sp*. HOT 177, Bergeyella sp. HOT 322, *Campylobacter concisus*, *Granulicatella adiacens*, *Kingella genus*, *Kingella dentifricans*, *Kingella oralis*, *Leptrotrichia hofstadii/Leptricihia sp*. HOT 223 or 234, *Streptococcus anginosus/S*.*gordonii*, and *Streptococcus sanguinis*, and those in the children having caries were: *Actinobaculum* sp. HOT 183, *Atopobium* genus, *Atopobium parvulum*, *Aggregatibacter sp*. HOT 513, *Streptococcus* genus, *Streptococcus sp*. HOT 431, *Streptococcus oralis*, and *S*.*mitis/Smitis bv2/ S*. *infantis* (Fig 9A and 9B).

## Discussion

The present study, monitored the oral microbiota, in the same children, at 3 months and 3 years of age, and, using DNA based methods, demonstrated that the overall species richness and taxa diversity in the mouth increases significantly during the first years of life. Most bacteria that colonize the oral cavity at 3 months of age persist at 3 years of age, but a few disappear, and a large number of different species are introduced during this time period. The major, and novel, finding, in this study was that, besides a more prevalent colonization with lactobacilli, the overall microbiota composition at 3 months of age was unrelated to caries development at a later age, whereas, several bacterial taxa in the oral biofilms of the 3 year olds could be linked to having caries or not at the same age.

The strengths of the present longitudinal study are that a specialist in pediatric dentistry, single-handedly, performed all clinical assessments, and that approximately 75% of the originally included infants could be seen at 3 years of age. The reason for the comparably high follow-up percentage is that, parents who were likely to leave the area, within the coming years, were not invited to participate in the study. Consequently, despite the low overall inclusion rate, the long-term compliance for those who consented to participate in the present study was high. This concept was chosen because one of the two towns where the participants were resident is a university town, comprising a large proportion of students of childbearing ages, who knew they would leave the area in the coming years. This may pose as a potential risk of selection bias in the present study cohort; however, a comparison between age, social status, and lifestyle factors, in a recent study involving early pregnant women living in the same area did not support the aforementioned notion [32].

The number of caries-affected children was small. This reflects that Sweden is a country with skewed caries prevalence in children and adolescents, *i*.*e*. a large portion being caries-free. The highest incidence is seen in children living in families with low socioeconomic status, and some immigrant groups. The incidence of caries in 3-year-olds in Sweden is reported to be 3 percent. The presently found prevalence is slightly higher compared to national data, but in accordance with other studies performed in the region [33, 34]. A potential limitation for caries detection was that the oral status did not justify a complementary X-ray in any child (see line 127). X-ray might have identified single children with initial approximal caries, but this was not doable due to tightly regulated criteria for X-ray in children by Swedish legislation. Based on that the children were included from the general population and the caries prevalence was as in the region combined with that an analysis method that is specially suited to find hidden structures was used, the present result should represent the situation in similar populations.

The sources of bacterial transmission to the infant’s mouth during the first few years of life include the mother’s vaginal, gut, and oral microbiota, the skin of the caretakers and siblings, breast milk, and other foods [33, 35]. In the present study group, taxa belonging to the *Escherichia*, *Staphylococcus*, and *Pseudomonas* genera were significantly more prevalent at 3 months of age than at 3 years of age, which may be a result of transmission through one or more of the aforementioned routes. Only 3 taxa (*Streptococcus*, *Veillonella*, and *Gemella)* in the core microbiome of the 3-month-old infants (present in all infants) persisted in all of the 3-year-olds. The *Streptococcus* and *Veillonella* genera produce lactate, and have been associated with ECC [22], whereas, *Gemella*, and other *Streptococcus* species such as *Streptococcus sanguinis*, have been found to be more prevalent in caries-free children [21]. The alterations in oral biofilm microbiota, between the 3-month-olds and the 3-year-olds in the present study, largely conform to that reported in the literature [36].

The proportion of children with lactobacilli in the mouth was 46% at 3 months, and 17% at 3 years of age and the species *L*.*crispatus* and *L*. *gasseri* tended to be more prevalent. We had previously reported the presence of lactobacilli in the mouths of infants who were exclusively breastfed, with *Lactobacillus gasseri* being the most dominant of the species [24]. Furthermore, *Lactobacillus gasseri* isolated from the infants was found to have antibacterial traits, suggesting that the early microbiota in the mouth may program for later healthy conditions. However, the present study does not support the hypothesis that the presence of *Lactobacillus gasseri*, or any other lactobacilli species at 3 to 4 months of age, has a major impact on the composition of the mature oral microbiota, or the possibility of remaining caries-free. On the contrary, the proportion of lactobacilli at 3 months of age was significantly higher in children who had caries at 3 years of age. This is contradictory to what has been reported for the gut microbiota, where the presence of lactobacilli has been reported to influence immune responses, nutrition, and overall wellbeing [37], but, is in line with the finding that supplementation with the probiotic *Lactobacillus paracasei* strain, F19, during early infancy, did not protect against caries development at a later age [38].

Mutans streptococci, especially *S*. *mutans*, were found to be more prevalent in children who had caries, in the present study. This is in agreement with several studies, which have reported the association between infections with mutans streptococci, mostly *S*. *mutans*, during the tooth eruption period, and early childhood caries (ECC) [39, 40]. However, similar to previous reports, not all children who developed caries were colonized with mutans streptococci (culture) or *S*. *mutans* (PCR), in the present study [13, 19].

There were two unexpected findings in the present study. The colonization of mutans streptococci (in culture) was more prevalent in girls, probably due to early tooth eruption [41, 42], thereby, providing the bacteria with longer time and more surface area for attachment and colonization. Furthermore, the time required for the development of caries is also increased. Notably, girls also tended to have more caries than boys, in the present study. Unfortunately, it was not possible to determine as to whether tooth eruption was earlier in the girls compared to boys, in this study, because 3 months is too early, whereas 3 years is too late to assess this phenomenon. The other unexpected finding was that children who had caries at 3 years of age, had accelerated growth compared to those who remained caries-free, i.e., they were significantly taller at 3 years of age, although the length at birth or at 3 months of age did not differ significantly. It is well known that children receiving balanced diets, and those with adequate protein intake have earlier growth spurts than the malnourished children. Moreover, malnourished children are also known to present with delayed teeth eruption. Children in Sweden are generally well nourished, and the present study included only those children who were healthy. Therefore, we presume that the accelerated growth in the 3 year olds with caries in this study is purely coincidental; however, we also believe that it should be followed-up in a future, larger study.

The present study identified taxa from *Actinomyces*, *Bergeyella*, *Campylobacter*, *Granulicatella*, *Kingella*, *Leptrotrichia*, *and Streptococcus genera*, to be associated with healthy teeth at 3 years of age. Several of these genera have previously been associated with healthy teeth (caries-free) in studies focusing on the whole microbiota. For example, *Actinomyces*, *Campylobacter*, *Leptotrichia*, *and Streptococcus* genera have been reported in Swedish adolescents living in the same area as those in the present study (Johansson *et al*., personal communication). Furthermore, *Campylobacter* [43], *Granulicatella* [44], *Kingella* [43], *Leptotrichia* [21], *and Streptococcus* (particularly *Streptococcus sanguinis)* [13, 39, 44, 45] have been associated with caries-free teeth in pre-school, and schoolchildren. However, comparisons with other studies is hampered by the fact that they are performed in different socio-economic conditions, different stages of the disease and different ages. It should also be kept in mind that to prove a causal or protective trait, identified species need to be tested in various types of experimental studies.

In contrast, the following taxa were associated with caries at 3 years of age: *Actinobaculum* sp. HOT 183, *Atopobium* genus, *Atopobium parvulum*, *Aggregatibacter sp*. HOT 513, *Streptococcus* genus, *Streptococcus sp*. HOT 431, *Streptococcus oralis*, and *S*.*mitis/Smitis bv2/ S*. *infantis*. Previous studies have linked the genera, *Atopobium* [45, 46], and *Streptococcus* [12, 21, 40, 45, 47, 48], with enamel or dentin caries in pre-school children, schoolchildren, and adolescents. Notably, the bacteria that were found to be overrepresented in caries may represent the causally related species, or those favored by the caries associated environment, such as, the species utilizing lactate for metabolism.

Based on the results in the present study, we conclude that both species richness and taxa diversity are significantly increased in the mouth, during the first years of life. Nevertheless, some taxa disappear with age. Furthermore, besides the more prevalent colonization of lactobacilli, the overall microbiota composition at 3 months of age was unrelated to caries development at a higher age. However, several taxa in the oral biofilms of the 3-year-olds could be associated with the presence or absence of caries at that age.

## Supporting Information

S1 Table Phyla and genera in 3 month and 3-year-old children. Proportion (%) children with detectable sequences in various phyla and genera, and mean prevalence of sequence proportions in these phyla and genera. Differences between age groups and groups with or without caries as 3 years of age were tested with Mann-Whitney test. P-values p≤0.008 are considered statistically significant.
(DOCX)

S2 Table Species/phylotypes in 3 month and 3-year-old children. Proportion (%) children with detectable sequences in various phyla and genera, and mean prevalence of sequence proportions in these phyla and genera. Differences between age groups were tested with Mann-Whitney test, whereas no univariate statistical analyses were performed between children with or without caries at 3 years of age (see methods section). P-values p≤0.008 are considered statistically significant.
(DOCX)

S3 Table Core microbiota and age associated species/phylotypes in 3-months and 3-years old children, respectively, in the MamBa study cohort by (A) pyrosequencing and (B) the HOMIM microarray. The oral microbiota was determined by (A) 454 FLX+ pyrosequencing and (B) the HOMIM microarray in samples from 3-months old infants and repeated when the child was 3-years of age. Core microbiota refers to bacteria detected in all children. Age characteristic species/phylotypes from (A) pyrosequencing and (B) HOMIM analyses were identified by PLS modeling with species/phylotypes as the independent block and age as the dependent variable. Species/phylotypes where the 95% CI did not include zero, i.e. statistically significant, are listed in alphabetical order.
(DOCX)
