# Supplementary material for: Maturation of Oral Microbiota in Children with or without Dental Caries
Source: PLoS One. 2015 May 28;10(5):e0128534. doi: 10.1371/journal.pone.0128534 (PMC4447273; doi:10.1371/journal.pone.0128534)
Supplement: S1 Table — Proportion (%) children with detectable sequences in various phyla and genera, and mean prevalence of sequence proportions in these phyla and genera. Differences between age groups and groups with or without caries as 3 years of age were tested with Mann-Whitney test. P-values p≤0.008 are considered statistically significant. (DOCX) [file pone.0128534.s001.docx]

**S1 Table. Phyla and genera in 3-month and 3-year old children**.

|  |  | Prevalence % yes detected | | |  | Prevalence mean % of all sequences^a^ | | | | | | |
| --- | --- | --- | --- | --- | --- | --- | --- | --- | --- | --- | --- | --- |
| Phylum | Genus | 3 months  n=22 | 3 years n=22 | p- value^b^ |  | 3 months  n=22 | 3 years n=22 | p -value^b^ |  | Caries free n=11 | Caries  n=11 | p -value^c^ |
| **Actinobacteria** |  | **90.9** | **100** | **0.148** |  | **2.8489** | **12.8204** | **<0.0001** |  | **14.8056** | **10.8252** | **0.478** |
|  | Actinobaculum | 0.0 | 40.9 | 0.001 |  | 0.0000 | 0.0153 | 0.001 |  | 0.0063 | 0.0243 | 0.438 |
|  | Actinomyces | 77.3 | 100 | 0.018 |  | 1.325 | 4.2431 | <0.0001 |  | 5.290 | 3.1872 | 0.065 |
|  | Atopobium | 59.1 | 40.9 | 0.228 |  | 0.1427 | 0.0108 | 0.090 |  | 0.0055 | 0.0160 | 0.562 |
|  | Bifidobacterium | 22.7 | 0.0 | 0.018 |  | 0.0947 | 0.0000 | 0.019 |  | 0.000 | 0.000 | 1.000 |
|  | Corynebacterium | 13.6 | 100.0 | <0.0001 |  | 0.0017 | 1.7585 | <0.0001 |  | 1.5988 | 1.9182 | 0.797 |
|  | Cryptobacterium | 0.0 | 4.5 | 0.312 |  | 0.0000 | 0.0006 | 0.317 |  | 0.0013 | 0.000 | 0.748 |
|  | Gardnerella | 4.5 | 0.0 | 0.312 |  | 0.0010 | 0.0000 | 0.317 |  | 0.0000 | 0.0000 | 1.000 |
|  | Kocuria | 9.1 | 0.0 | 0.148 |  | 0.0006 | 0.0000 | 0.153 |  | 0.0000 | 0.0000 | 1.000 |
|  | Parascardovia | 4.5 | 0.0 | 0.312 |  | 0.0007 | 0.0000 | 0.317 |  | 0.000 | 0.0000 | 1.000 |
|  | Propionibacterium | 0.0 | 13.6 | 0.073 |  | 0.0000 | 0.0009 | 0.076 |  | 0.0000 | 0.0018 | 0.300 |
|  | Rothia | 86.4 | 100 | 0.072 |  | 1.2736 | 6.7878 | <0.0001 |  | 7.9011 | 5.6745 | 0.217 |
|  | Sanguibacter | 9.1 | 0.0 | 0.148 |  | 0.0005 | 0.0000 | 0.153 |  | 0.0000 | 0.0000 | 1.000 |
|  | Scardovia | 4.5 | 13.6 | 0.294 |  | 0.0084 | 0.0034 | 0.335 |  | 0.0026 | 0.0032 | 0.797 |
| **Bacteroidetes** |  | **100** | **100** | **1.0** |  | **0.7049** | **5.6222** | **<0.0001** |  | **5.2680** | **5.9718** | **0.438** |
|  | Alloprevotella | 45.5 | 100 | <0.0001 |  | 0.4396 | 0.7893 | <0.0001 |  | 0.3964 | 1.1822 | 0.562 |
|  | Bergeyella | 40.9 | 100 | <0.0001 |  | 0.0110 | 0.2340 | <0.0001 |  | 0.2818 | 0.1862 | 0.401 |
|  | Capnocytophaga | 27.3 | 100 | <0.0001 |  | 0.0051 | 2.5330 | <0.0001 |  | 2.4429 | 2.6230 | 0.652 |
|  | Porphyromonas | 36.4 | 100.0 | <0.0001 |  | 0.0213 | 1.1530 | <0.0001 |  | 1.0923 | 1.208 | 0.562 |
|  | Prevotella | 86.4 | 95.5 | 0.294 |  | 0.2279 | 0.9129 | <0.0001 |  | 1.0534 | 0.7724 | 0.699 |
| **Firmicutes** |  | **100** | **100** | **1.0** |  | **94.6335** | **59.6530** | **<0.0001** |  | **58.7103** | **61.5325** | **0.438** |
|  | Abiotrophia | 13.6 | 100 | <0.0001 |  | 0.0010 | 0.8001 | <0.0001 |  | 0.8415 | 0.7588 | 0.847 |
|  | Catonella | 4.5 | 18.2 | 0.154 |  | 0.0001 | 0.0031 | 0.136 |  | 0.0036 | 0.0025 | 1.000 |
|  | Clostridiales | 0.0 | 36.4 | 0.002 |  | 0.0000 | 0.0131 | 0.002 |  | 0.0098 | 0.0165 | 0.478 |
|  | Dialister | 0.0 | 27.3 | 0.008 |  | 0.0000 | 0.0195 | 0.009 |  | 0.0292 | 0.0097 | 0.949 |
|  | Dolosigranulum | 4.5 | 4.5 | 1.000 |  | 0.0003 | 0.0004 | 0.974 |  | 0.0007 | 0.000 | 0.748 |
|  | Eubacterium | 4.5 | 72.9 | <0.0001 |  | 0.0003 | 0.0824 | <0.0001 |  | 0.1079 | 0.0569 | 0.116 |
|  | Finegoldia | 9.1 | 0.0 | 0.148 |  | 0.0007 | 0.0000 | 0.153 |  | 0.0000 | 0.0000 | 1.000 |
|  | Gemella | 100.0 | 100.0 | 1.000 |  | 5.2410 | 6.8414 | 0.121 |  | 7.0222 | 6.6606 | 0.949 |
|  | Granulicatella | 90.9 | 100.0 | 0.148 |  | 0.8571 | 3.7184 | <0.0001 |  | 3.5552 | 3.8815 | 0.056 |
|  | Johnsonella | 0.0 | 4.5 | 0.312 |  | 0.0000 | 0.0010 | 0.317 |  | 0.0020 | 0.0000 | 0.748 |
|  | Lachnoanaerobaculum | 13.6 | 36.4 | 0.082 |  | 0.0104 | 0.0055 | 0.090 |  | 0.0025 | 0.0085 | 0.401 |
|  | Lachnospiraceae | 18.2 | 68.2 | 0.001 |  | 0.0111 | 0.0457 | 0.001 |  | 0.452 | 0.463 | 0.949 |
|  | Lactobacillus | 36.4 | 9.1 | 0.031 |  | 0.0343 | 0.0022 | 0.038 |  | 0.0007 | 0.0038 | 1.00 |
|  | Megasphaera | 13.6 | 18.2 | 0.680 |  | 0.0013 | 0.0020 | 0.622 |  | 0.0021 | 0.0019 | 0.562 |
|  | Mitsuokella | 4.5 | 0.0 | 0.312 |  | 0.0014 | 0.0000 | 0.317 |  | 0.0000 | 0.0000 | 1.000 |
|  | Mogibacterium | 0.0 | 27.3 | 0.008 |  | 0.0000 | 0.0087 | 0.009 |  | 0.0079 | 0.0095 | 1.000 |
|  | Moryella | 9.1 | 18.2 | 0.380 |  | 0.0011 | 0.0063 | 0.366 |  | 0.0005 | 0.0121 | 0.438 |
|  | Oribacterium | 9.1 | 68.2 | <0.0001 |  | 0.0041 | 0.0446 | <0.0001 |  | 0.0262 | 0.0630 | 0.365 |
|  | Parvimonas | 0.0 | 18.2 | 0.036 |  | 0.0000 | 0.0262 | 0.038 |  | 0.0484 | 0.0039 | 0.478 |
|  | Peptococcus | 0.0 | 9.1 | 0.148 |  | 0.0000 | 0.0006 | 0.153 |  | 0.0000 | 0.0012 | 0.478 |
|  | Peptoniphilus | 4.5 | 0.0 | 0.312 |  | 0.0003 | 0.0000 | 0.317 |  | 0.0000 | 0.0000 | 1.000 |
|  | Peptostreptococcus | 4.5 | 30.9 | 0.004 |  | 0.0004 | 0.0119 | 0.004 |  | 0.0091 | 0.148 | 0.898 |
|  | Selenomonas | 18.2 | 90.9 | <0.0001 |  | 0.0014 | 0.7237 | <0.0001 |  | 1.0591 | 0.3882 | 0.606 |
|  | Staphylococcus | 63.6 | 18.2 | 0.002 |  | 0.0604 | 0.0051 | 0.003 |  | 0.0079 | 0.0024 | 0.949 |
|  | Streptococcus | 100.0 | 100.0 | 1.000 |  | 81.7798 | 44.8429 | 0.076 |  | 42.7397 | 46.9262 | 0.171 |
|  | Veillonella | 100.0 | 100.0 | 1.000 |  | 6.6270 | 2.4482 | 0.197 |  | 2.7821 | 2.1143 | 0.478 |
| **Fusobacteria** |  | **31.8** | **100** | **<0.0001** |  | **0.1276** | **2.7252** | **<0.0001** |  | **3.1084** | **2.4022** | **0.748** |
|  | Fusobacterium | 27.3 | 100 | <0.0001 |  | 0.0135 | 0.9043 | <0.0001 |  | 0.8530 | 0.9557 | 0.797 |
|  | Leptotrichia | 18.2 | 100.0 | <0.0001 |  | 0.1141 | 1.8509 | <0.0001 |  | 2.2554 | 1.4465 | 0.438 |
| **Proteobacteria** |  | **100** | **100** | **1.0000** |  | **1.6663** | **19.0043** | **<0.0001** |  | **18.3551** | **19.6646** | **0.652** |
|  | Acinetobacter | 9.1 | 0.0 | 0.148 |  | 0.0005 | 0.0000 | 0.153 |  | 0.000 | 0.000 | 1.00 |
|  | Aggregatibacter | 18.2 | 100.0 | <0.0001 |  | 0.0010 | 2.4908 | <0.0001 |  | 1.8714 | 3.1102 | 0.193 |
|  | Burkholderia | 4.5 | 4.5 | 1.000 |  | 0.0003 | 0.0007 | 0.974 |  | 0.000 | 0.0014 | 0.748 |
|  | Campylobacter | 0.0 | 90.9 | <0.0001 |  | 0.0000 | 0.0479 | <0.0001 |  | 0.0416 | 0.0542 | 0.599 |
|  | Cardiobacterium | 0.0 | 22.7 | 0.018 |  | 0.0000 | 0.0087 | 0.019 |  | 0.0162 | 0.0012 | 0.606 |
|  | Delftia | 13.6 | 0.0 | 0.073 |  | 0.0010 | 0.0000 | 0.076 |  | 0.0000 | 0.0000 | 1.000 |
|  | Eikenella | 0.0 | 77.2 | <0.0001 |  | 0.0000 | 0.0415 | <0.0001 |  | 0.0271 | 0.0559 | 0.401 |
|  | Enterobacter | 13.6 | 27.3 | 0.262 |  | 0.0013 | 0.0045 | 0.231 |  | 0.0010 | 0.0080 | 0.316 |
|  | Escherichia | 50.0 | 4.5 | 0.001 |  | 0.0053 | 0.0005 | 0.001 |  | 0.000 | 0.0010 | 0.348 |
|  | Haemophilus | 62.7 | 100.0 | 0.008 |  | 0.6597 | 7.3337 | <0.0001 |  | 6.9854 | 7.6821 | 0.652 |
|  | Kingella | 9.1 | 100.0 | <0.0001 |  | 0.0005 | 0.7979 | <0.0001 |  | 1.0512 | 0.5566 | 0.040 |
|  | Klebsiella | 9.1 | 0.0 | 0.148 |  | 0.0013 | 0.0005 | 0.153 |  | 0.0000 | 0.0000 | 1.000 |
|  | Lautropia | 4.5 | 100.0 | <0.0001 |  | 0.0003 | 1.7414 | <0.0001 |  | 1.5149 | 1.9678 | 1.000 |
|  | Moraxella | 9.1 | 0.0 | 0.148 |  | 0.0017 | 0.0000 | 0.153 |  | 0.0000 | 0.000 | 1.000 |
|  | Neisseria | 86.4 | 100.0 | 0.073 |  | 0.9665 | 6.5277 | <0.0001 |  | 6.8332 | 6.2222 | 1.000 |
|  | Ochrobactrum | 4.5 | 0.0 | 0.312 |  | 0.0003 | 0.0000 | 0.317 |  | 0.0000 | 0.0000 | 1.000 |
|  | Ottowia | 0.0 | 4.5 | 0.312 |  | 0.0000 | 0.0016 | 0.317 |  | 0.0032 | 0.0000 | 0.748 |
|  | Pseudomonas | 86.4 | 27.3 | <0.0001 |  | 0.0252 | 0.0038 | <0.0001 |  | 0.0060 | 0.0016 | 0.847 |
|  | Ralstonia | 9.1 | 9.1 | 1.000 |  | 0.0003 | 0.0011 | 0.925 |  | 0.0023 | 0.0000 | 0.478 |
|  | Simonsiella | 0.0 | 13.6 | 0.073 |  | 0.0000 | 0.0020 | 0.076 |  | 0.0016 | 0.0024 | 0.797 |
|  | Stenotrophomonas | 18.2 | 0.0 | 0.026 |  | 0.0011 | 0.0000 | 0.038 |  | 0.0000 | 0.000 | 1.000 |
| **SR1** | **SR1[G-1]** | **0.0** | **36.4** | **0.002** |  | **0.0000** | **0.0098** | **0.002** |  | **0.0088** | **0.0109** | **0.699** |
| **TM7** | **TM7[G-]** | **13.6** | **77.3** | **<0.0001** |  | **0.0088** | **0.0645** | **<0.0001** |  | **0.0673** | **0.0617** | **0.606** |

1. Mean prevalence of sequences are presented since many genera were present in less than 50% in both age groups and hence medians equal to zero. Generally medians were lower than mean values.
2. P-value for test between age groups.
3. P-value for test between 3 year olds with or without caries.
