# Supplementary material for: Maturation of Oral Microbiota in Children with or without Dental Caries
Source: PLoS One. 2015 May 28;10(5):e0128534. doi: 10.1371/journal.pone.0128534 (PMC4447273; doi:10.1371/journal.pone.0128534)
Supplement: S2 Table — Proportion (%) children with detectable sequences in various phyla and genera, and mean prevalence of sequence proportions in these phyla and genera. Differences between age groups were tested with Mann-Whitney test, whereas no univariate statistical analyses were performed between children with or without caries at 3 years of age (see methods section). P-values p≤0.008 are considered statistically significant. (DOCX) [file pone.0128534.s002.docx]

**S2 Table. Species/phylotypes in the MamBa cohort.**

|  | Comparison by age | | | | | |  | Comparison by caries status at 3 years of age | | | |
| --- | --- | --- | --- | --- | --- | --- | --- | --- | --- | --- | --- |
|  | % with species | | | Mean prevalence in children with species | | |  | % with species | | Mean prevalence in children with species | |
|  | 3 months  n=22 | 3 years n=22 | p -value^b^ | 3 months  n=22 | 3 years n=22 | p -value^b^ |  | caries-free n=11 | caries  n=11 | caries- free  n=11 | caries  n=11 |
| *Abiotrophia defectiva* | 13.6 | 100.0 | <0.001 | 0.0010 | 0.8001 | <0.001 |  | 100 | 100 | 0.842 | 0.759 |
| *Acinetobacter sp. HOT 408* | 9.1 | 0.0 |  | 0.0005 | 0.0000 |  |  | 0.0 | 0.0 | 0.000 | 0.000 |
| *Actinobaculum sp. HOT 183* | 0.0 | 40.9 | 0.001 | 0.0000 | 0.0153 | 0.024 |  | 36.4 | 45.5 | 0.006 | 0.024 |
| *Actinomyces graevenitzii* | 45.5 | 54.5 |  | 0.5227 | 0.0517 |  |  | 45.5 | 63.6 | 0.042 | 0.061 |
| *Actinomyces johnsonii sp. HOT 171_849* | 4.5 | 100.0 | <0.001 | 0.0003 | 0.8440 |  |  | 100.0 | 100.0 | 0.684 | 1.000 |
| *Actinomyces massiliensis* | 0.0 | 9.1 |  | 0.0000 | 0.0024 | <0.001 |  | 18.2 | 0.0 | 0.005 | 0.000 |
| *Actinomyces naeslundii* | 18.2 | 18.2 |  | 0.0470 | 0.0049 |  |  | 9.1 | 27.3 | 0.001 | 0.009 |
| *Actinomyces sp. HOT 71 / A. naeslundii II* | 4.5 | 13.6 |  | 0.0001 | 0.0010 |  |  | 18.2 | 9.1 | 0.001 | 0.009 |
| *Actinomyces oris* | 13.6 | 100.0 | <0.001 | 0.0014 | 1.4326 | <0.001 |  | 100.0 | 100.0 | 1.914 | 0.952 |
| *Actinomyces sp. HOT 171* | 0.0 | 86.4 | <0.001 | 0.0000 | 0.1049 | 0.039 |  | 90.9 | 81.8 | 0.131 | 0.079 |
| *Actinomyces sp. HOT 172* | 22.7 | 50.0 |  | 0.0542 | 0.0308 |  |  | 45.5 | 54.5 | 0.015 | 0.047 |
| *Actinomyces sp. HOT 175* | 0.0 | 50.0 | <0.001 | 0.0000 | 0.0532 | 0.034 |  | 36.4 | 63.6 | 0.071 | 0.036 |
| *Actinomyces sp. HOT 177* | 9.1 | 95.5 | <0.001 | 0.0020 | 1.0981 | <0.001 |  | 100.0 | 90.9 | 1.509 | 0.688 |
| *Actinomyces sp. HOT 178* | 0.0 | 31.9 | 0.004 | 0.0000 | 0.1656 |  |  | 36.4 | 27.3 | 0.306 | 0.025 |
| *Actinomyces sp. HOT 180* | 72.7 | 100.0 | 0.008 | 0.3235 | 0.3550 |  |  | 100.0 | 100.0 | 0.486 | 0.224 |
| *Actinomyces sp. HOT 181* | 68.2 | 50.0 |  | 0.8804 | 0.0168 |  |  | 54.5 | 45.5 | 0.016 | 0.013 |
| *Actinomyces sp. HOT 877* | 0.0 | 18.2 | 0.036 | 0.0000 | 0.0072 |  |  | 27.3 | 9.1 | 0.013 | 0.001 |
| *Aggregatibacter actinomycetemcomitans* | 0.0 | 4.5 |  | 0.0000 | 0.0009 |  |  | 0.0 | 9.1 | 0.000 | 0.002 |
| *Aggregatibacter aphrophilus* | 0.0 | 63.6 | <0.001 | 0.0000 | 0.6666 |  |  | 63.6 | 63.6 | 0.070 | 1.263 |
| *Aggregatibacter paraphrophilus* | 0.0 | 95.5 | <0.001 | 0.0000 | 0.4071 | 0.001 |  | 100.0 | 90.9 | 0.454 | 0.361 |
| *Aggregatibacter segnis* | 4.5 | 40.9 | 0.004 | 0.0002 | 0.1013 | 0.029 |  | 36.4 | 45.5 | 0.071 | 0.131 |
| *Aggregatibacter sp. HOT 458* | 4.5 | 81.8 | <0.001 | 0.0003 | 0.4426 | <0.001 |  | 90.9 | 72.7 | 0.483 | 0.403 |
| *Aggregatibacter sp. HOT 513* | 0.0 | 54.5 | <0.001 | 0.0000 | 0.2238 | 0.035 |  | 45.5 | 63.6 | 0.030 | 0.418 |
| *Aggregatibacter sp. HOT 898* | 0.0 | 72.7 | <0.001 | 0.0000 | 0.3997 | 0.014 |  | 63.6 | 81.8 | 0.490 | 0.315 |
| *Alloprevotella sp. HOT 302 / A. rava* | 0.0 | 4.5 |  | 0.0000 | 0.0005 |  |  | 9.1 | 0.0 | 0.001 | 0.000 |
| *Alloprevotella sp. HOT 308* | 4.5 | 45.5 | 0.002 | 0.0003 | 0.0055 | 0.003 |  | 45.5 | 45.5 | 0.007 | 0.004 |
| *Alloprevotella sp. HOT 473* | 45.5 | 95.5 | <0.001 | 0.4393 | 0.7818 |  |  | 100.0 | 90.9 | 0.386 | 1.178 |
| *Alloprevotella tannerae* | 0.0 | 4.5 |  | 0.0000 | 0.0014 |  |  | 9.1 | 0.0 | 0.003 | 0.000 |
| *Atopobium parvulum* | 59.1 | 40.9 |  | 0.1424 | 0.0105 |  |  | 36.4 | 45.5 | 0.005 | 0.016 |
| *Atopobium rimae / Parvulum sp. HOT 199* | 4.5 | 4.5 |  | 0.0003 | 0.0003 |  |  | 9.1 | 0.0 | 0.001 | 0.000 |
| *Bergeyella sp. HOT 322* | 40.9 | 100.0 | <0.001 | 0.0110 | 0.1991 | <0.001 |  | 100.0 | 100.0 | 0.235 | 0.163 |
| *Bergeyella sp. HOT 900* | 0.0 | 45.5 | <0.001 | 0.0000 | 0.0133 | 0.008 |  | 45.5 | 45.5 | 0.012 | 0.015 |
| *Bergeyella sp. HOT 907* | 0.0 | 54.5 | <0.001 | 0.0000 | 0.0215 | 0.043 |  | 72.7 | 36.4 | 0.035 | 0.008 |
| *Bifidobacterium scardovii* | 4.5 | 0.0 |  | 0.4590 | 0.0000 |  |  | 0.0 | 0.0 | 0.000 | 0.000 |
| *Bifidobacterium breve* | 13.6 | 0.0 |  | 0.0480 | 0.0000 |  |  | 0.0 | 0.0 | 0.000 | 0.000 |
| *Bifidobacterium longum* | 9.1 | 0.0 |  | 0.0008 | 0.0000 |  |  | 0.0 | 0.0 | 0.000 | 0.000 |
| *Burkholderia cepacia* | 4.5 | 4.5 |  | 0.0003 | 0.0070 |  |  | 0.0 | 9.1 | 0.000 | 0.001 |
| *Campylobacter concisus* | 31.8 | 90.9 | <0.001 | 0.0210 | 0.0765 | 0.006 |  | 90.9 | 90.9 | 0.073 | 0.080 |
| *Campylobacter rectus* | 0.0 | 86.4 | <0.001 | 0.0000 | 0.0421 | 0.003 |  | 90.9 | 81.8 | 0.038 | 0.046 |
| *Campylobacter showae / C. rectus* | 0.0 | 31.8 | 0.004 | 0.0000 | 0.0058 | 0.013 |  | 18.2 | 45.5 | 0.004 | 0.008 |
| *Capnocytophaga granulosa* | 0.0 | 68.2 | <0.001 | 0.0000 | 0.0615 | 0.007 |  | 63.6 | 72.7 | 0.049 | 0.074 |
| *Capnocytophaga leadbetteri* | 4.5 | 95.5 | <0.001 | 0.0005 | 0,1863 | <0.001 |  | 90.9 | 100.0 | 0.151 | 0.221 |
| *Capnocytophaga sp. HOT 326* | 0.0 | 72.7 | <0.001 | 0.0000 | 0.0818 | 0.029 |  | 72.7 | 72.7 | 0.117 | 0.046 |
| *Capnocytophaga sp. HOT 332* | 0.0 | 36.4 | 0.002 | 0.0000 | 0.0205 | 0.023 |  | 36.4 | 36.4 | 0.025 | 0.016 |
| *Capnocytophaga sp. HOT 336* | 0.0 | 9.1 |  | 0.0000 | 0.0021 |  |  | 0.0 | 18.2 | 0.000 | 0.004 |
| *Capnocytophaga sp. HOT 863* | 0.0 | 4.5 |  | 0.0000 | 0.0280 |  |  | 9.1 | 0.0 | 0.006 | 0.000 |
| *Capnocytophaga sp. HOT 864* | 4.5 | 100.0 | <0.001 | 0.0003 | 0.2885 | <0.001 |  | 100.0 | 100.0 | 0.332 | 0.244 |
| *Capnocytophaga sp. HOT 878* | 0.0 | 13.6 | 0.073 | 0.0000 | 0.0361 |  |  | 9.1 | 18.2 | 0.008 | 0.065 |
| *Capnocytophaga sp. HOT 901* | 0.0 | 13.6 |  | 0.0000 | 0.0039 |  |  | 9.1 | 18.2 | 0.006 | 0.002 |
| *Capnocytophaga sp. HOT 902_336* | 0.0 | 50.0 | <0.001 | 0.0000 | 0.0199 | 0.004 |  | 63.6 | 36.4 | 0.028 | 0.011 |
| *Capnocytophaga sp. HOT 903_412* | 0.0 | 22.7 | 0.018 | 0.0000 | 0.0087 |  |  | 27.3 | 18.2 | 0.005 | 0.012 |
| *Capnocytophaga sputigena* | 27.3 | 100.0 | <0.001 | 0.0043 | 1.8176 | <0.001 |  | 100.0 | 100.0 | 1.710 | 1.925 |
| *Catonella morbi sp. HOT 164_165* | 0.0 | 9.1 |  | 0.0000 | 0.0024 |  |  | 9.1 | 9.1 | 0.003 | 0.002 |
| *Clostridiales [F-2][G-1] sp. HOT 075* | 0.0 | 36.4 | 0.002 | 0.0000 | 0.0119 | 0.024 |  | 27.3 | 45.5 | 0.010 | 0.141 |
| *Clostridiales [F-2][G-2] sp. HOT 085* | 0.0 | 4.5 |  | 0.0000 | 0.0012 |  |  | 0.0 | 9.1 | 0.000 | 0.002 |
| *Corynebacterium durum* | 4.5 | 100.0 | <0.001 | 0.0006 | 1.7517 | 0.001 |  | 100.0 | 100.0 | 1.585 | 1.918 |
| *Cryptobacterium curtum* | 0.0 | 4.5 |  | 0.0000 | 0.0006 |  |  | 9.1 | 0.0 | 0.001 | 0.000 |
| *Delftia acidovorans* | 13.6 | 0.0 |  | 0.0010 | 0.0000 |  |  | 0.0 | 0.0 | 0.000 | 0.000 |
| *Dialister invisus* | 0.0 | 27.3 | 0.008 | 0.0000 | 0.0195 |  |  | 27.3 | 27.3 | 0.029 | 0.009 |
| *Dolosigranulum pigrum* | 4.5 | 4.5 |  | 0.0003 | 0.0004 |  |  | 9.1 | 0.0 | 0.007 | 0.000 |
| *Escherichia coli* | 50.0 | 4.5 | 0.001 | 0.0053 | 0.0005 | 0.006 |  | 0.0 | 9.1 | 0.000 | 0.001 |
| *Eubacterium [XI][G-1] sulci* | 0.0 | 18.2 | 0.036 | 0.0000 | 0.0039 |  |  | 27.3 | 9.1 | 0.004 | 0.003 |
| *Eubacterium [XI][G-7] yurii* | 0.0 | 27.3 | 0.008 | 0.0000 | 0.0035 | 0.044 |  | 18.2 | 36.4 | 0.040 | 0.003 |
| *Eubacterium [XIVa][G-1] saburreum* | 4.5 | 54.5 | <0.001 | 0.0003 | 0.0749 | 0.010 |  | 63.6 | 45.5 | 0.099 | 0.055 |
| *Fusobacterium naviforme* | 0.0 | 22.7 | 0.018 | 0.0000 | 0.0045 |  |  | 27.3 | 18.2 | 0.007 | 0.003 |
| *Fusobacterium nucleatum ss polymorphum* | 22.7 | 100.0 | <0.001 | 0.0026 | 0.7345 | <0.001 |  | 100.0 | 100.0 | 0.716 | 0.753 |
| *Fusobacterium nucleatum ss vincentii* | 0.0 | 22.7 | 0.018 | 0.0000 | 0.0031 | 0.049 |  | 36.4 | 9.1 | 0.005 | 0.002 |
| *Fusobacterium periodonticum* | 9.1 | 77.3 | <0.001 | 0.0030 | 0.1159 | 0.019 |  | 81.8 | 72.7 | 0.055 | 0.176 |
| *Gemella haemolysans* | 100.0 | 100.0 |  | 5.1851 | 6.3784 |  |  | 100.0 | 100.0 | 6.527 | 6.230 |
| *Gemella morbillorum* | 0.0 | 72.7 | <0.001 | 0.0000 | 0.3613 | 0.014 |  | 72.7 | 72.7 | 0.349 | 0.374 |
| *Gemella sanguinis* | 59.1 | 95.5 | 0.004 | 0.0559 | 0.1004 |  |  | 100.0 | 90.9 | 0.145 | 0.056 |
| *Granulicatella adiacens [para-adiacens]* | 50.0 | 100.0 | <0.001 | 0.2477 | 2.1192 | <0.001 |  | 100.0 | 100.0 | 2.356 | 1.882 |
| *Granulicatella elegans* | 72.7 | 100.0 | 0.008 | 0.6094 | 1.5989 | 0.017 |  | 100.0 | 100.0 | 1.199 | 1.999 |
| *Haemophilus haemolyticus* | 40.9 | 81.8 | 0.005 | 0.2560 | 0.2951 | 0.001 |  | 81.9 | 81.9 | 0.259 | 0.331 |
| *Haemophilus influenzae* | 100.0 | 100.0 |  | 0.0000 | 0.1736 | 0.039 |  | 54.5 | 72.7 | 0.134 | 0.213 |
| *Haemophilus parainfluenzae* | 40.9 | 100.0 | <0.001 | 0.2902 | 5.7778 | <0.001 |  | 100.0 | 100.0 | 5.643 | 5.912 |
| *Haemophilus sp. HOT 035* | 9.1 | 86.4 | <0.001 | 0.0029 | 0.4173 | 0.003 |  | 72.7 | 100.0 | 0.464 | 0.370 |
| *Haemophilus sp. HOT 036* | 63.6 | 100.0 | 0.002 | 0.3203 | 0.4669 |  |  | 100.0 | 100.0 | 0.367 | 0.567 |
| *Haemophilus sp. HOT 908* | 36.4 | 90.9 | <0.001 | 0.0207 | 0.2025 | 0.004 |  | 81.8 | 100.0 | 0.117 | 0.288 |
| *Johnsonella ignava* | 0.0 | 4.5 |  | 0.0000 | 0.0010 |  |  | 9.1 | 0.0 | 0.002 | 0.000 |
| *Kingella denitrificans* | 0.0 | 50.0 | <0.001 | 0.0000 | 0.2717 | 0.001 |  | 90.9 | 100.0 | 0.223 | 0.320 |
| *Kingella oralis* | 9.1 | 100.0 | <0.001 | 0.0005 | 0.4776 | <0.001 |  | 100.0 | 100.0 | 0.749 | 0.207 |
| *Klebsiella pneumoniae* | 9.1 | 0.0 |  | 0.0013 | 0.0000 |  |  | 0.0 | 0.0 | 0.000 | 0.000 |
| *Lachnoanaerobaculum orale* | 13.6 | 36.4 |  | 0.0104 | 0.0055 |  |  | 27.3 | 45.5 | 0.003 | 0.009 |
| *Lachnospiraceae [G-2] sp. HOT 088* | 0.0 | 27.3 | 0.008 | 0.0000 | 0.1320 |  |  | 36.4 | 18.2 | 0.021 | 0.005 |
| *Lachnospiraceae [G-5] sp. HOT 455* | 0.0 | 4.5 |  | 0.0000 | 0.0005 |  |  | 0.0 | 9.1 | 0.000 | 0.001 |
| *Lactobacillus crispatus* | 27.7 | 0.0 | 0.018 | 0.0017 | 0.0000 |  |  | 100.0 | 100.0 | 0.000 | 0.000 |
| *Lactobacillus fermentum* | 4.5 | 9.1 |  | 0.0001 | 0.0008 |  |  | 9.1 | 9.1 | 0.001 | 0.001 |
| *Lactobacillus gasseri* | 27.3 | 4.5 | 0.039 | 0.0325 | 0.0014 |  |  | 0.0 | 9.1 | 0.000 | 0.003 |
| *Lautropia mirabilis* | 4.5 | 100.0 | <0.001 | 0.0003 | 1.7412 | <0.001 |  | 100.0 | 100.0 | 1.515 | 1.968 |
| *Leptotrichia goodfellowii* | 0.0 | 50.0 | <0.001 | 0.0000 | 0.0338 | 0.019 |  | 54.5 | 45.5 | 0.048 | 0.019 |
| *Leptotrichia hofstadii sp. HOT 909* | 4.5 | 81.9 | <0.001 | 0.0003 | 0.2441 | <0.001 |  | 90.9 | 72.7 | 0.250 | 0.238 |
| *Leptotrichia shahii* | 4.5 | 77.3 | <0.001 | 0.0052 | 0.5426 | 0.028 |  | 81.8 | 72.7 | 0.864 | 0.221 |
| *Leptotrichia sp. HOT 215* | 9.1 | 86.4 | <0.001 | 0.0056 | 0.0488 | 0.023 |  | 90.9 | 81.8 | 0.021 | 0.076 |
| *Leptotrichia sp. HOT 218* | 0.0 | 9.1 |  | 0.0000 | 0.0008 |  |  | 9.1 | 9.1 | 0.001 | 0.001 |
| *Leptotrichia sp. HOT 219* | 0.0 | 36.4 | 0.002 | 0.0000 | 0.0265 | 0.036 |  | 27.3 | 45.5 | 0.024 | 0.029 |
| *Leptotrichia sp. HOT 221* | 9.1 | 63.6 | <0.001 | 0.1005 | 0.0529 |  |  | 81.8 | 45.5 | 0.027 | 0.079 |
| *Leptotrichia sp. HOT 392* | 0.0 | 90.9 | <0.001 | 0.0000 | 0.1028 | <0.001 |  | 90.9 | 90.9 | 0.078 | 0.128 |
| *Leptotrichia sp. HOT 879* | 0.0 | 54.5 | <0.001 | 0.0000 | 0.1604 | 0.032 |  | 54.5 | 54.5 | 0.140 | 0.182 |
| *Leptotrichia sp. HOT 212_217* | 4.5 | 95.5 | <0.001 | 0.0008 | 0.5630 | 0.002 |  | 90.9 | 100.0 | 0.749 | 0.377 |
| *Leptotrichia sp. HOT 462_417* | 9.1 | 54.5 | 0.001 | 0.0017 | 0.0284 |  |  | 54.5 | 54.5 | 0.012 | 0.045 |
| *Leptotrichia sp. HOT 225 / L. buccalis* | 0.0 | 77.3 | <0.001 | 0.0000 | 0.0316 | 0.001 |  | 63.6 | 90.9 | 0.037 | 0.026 |
| *Mitsuokella sp. HOT 521* | 4.5 | 0.0 |  | 0.0015 | 0.0000 |  |  | 0.0 | 0.0 | 0.000 | 0.000 |
| *Mogibacterium neglectum / M. vesucum* | 0.0 | 27.3 | 0.008 | 0.0000 | 0.0087 |  |  | 27.3 | 27.3 | 0.008 | 0.010 |
| *Moraxella osloensis* | 9.1 | 0.0 |  | 0.0017 | 0.0000 |  |  | 0.0 | 0.0 | 0.000 | 0.000 |
| *Moryella sp. HOT 419* | 9.1 | 18.2 |  | 0.0011 | 0.0063 |  |  | 9.1 | 27.3 | 0.001 | 0.012 |
| *Neisseria elongata* | 0.0 | 100.0 | <0.001 | 0.0000 | 0.2638 |  |  | 90.9 | 100.0 | 0.147 | 0.381 |
| *Neisseria flavescens* | 72.7 | 100.0 | <0.001 | 0.9291 | 0.8352 |  |  | 100.0 | 100.0 | 1.079 | 0.591 |
| *Neisseria mucosa / N. Flava* | 36.4 | 100.0 | <0.001 | 0.0211 | 4.7158 | <0.001 |  | 100.0 | 100.0 | 4.728 | 4.703 |
| *Neisseria sicca* | 0.0 | 38.8 | 0.004 | 0.0000 | 0.0080 |  |  | 27.3 | 36.4 | 0.013 | 0.003 |
| *Neisseria subflava* | 9.1 | 77.3 | <0.001 | 0.0030 | 0.2556 | 0.042 |  | 72.7 | 81.8 | 0.379 | 0.134 |
| *Ochrobactrum anthropi* | 4.5 | 0.0 |  | 0.0003 | 0.0000 |  |  | 0.0 | 0.0 | 0.000 | 0.000 |
| *Oribacterium sp. HOT 108* | 9.1 | 68.2 | <0.001 | 0.0041 | 0.0430 |  |  | 81.8 | 54.5 | 0.023 | 0.063 |
| *Oribacterium sp. HOT 78_372* | 0.0 | 4.5 |  | 0.0000 | 0.0016 |  |  | 9.1 | 0.0 | 0.003 | 0.000 |
| *Ottowia sp. HOT 894* | 0.0 | 4.5 |  | 0.0000 | 0.0016 |  |  | 9.1 | 0.0 | 0.003 | 0.000 |
| *Parascardovia denticolens* | 4.5 | 0.0 |  | 0.0008 | 0.0000 |  |  | 0.0 | 0.0 | 0.000 | 0.000 |
| *Parvimonas micra* | 0.0 | 9.1 |  | 0.0000 | 0.0058 |  |  | 18.2 | 0.0 | 0.012 | 0.000 |
| *Parvimonas sp. HOT 110* | 0.0 | 13.6 |  | 0.0000 | 0.025 |  |  | 18.2 | 9.1 | 0.002 | 0.003 |
| *Parvimonas sp. HOT 110_393* | 0.0 | 18.2 | 0.036 | 0.0000 | 0.0179 |  |  | 27.3 | 9.1 | 0.035 | 0.001 |
| *Peptococcus sp. HOT 167* | 0.0 | 9.1 |  | 0.0000 | 0.0006 |  |  | 0.0 | 18.2 | 0.000 | 0.001 |
| *Peptostreptococcus stomatis* | 4.5 | 40.9 | 0.004 | 0.0004 | 0.0119 |  |  | 45.5 | 36.4 | 0.009 | 0.015 |
| *Porphyromonas catoniae* | 0.0 | 86.4 | <0.001 | 0.0000 | 0.2397 | <0.001 |  | 90.9 | 81.8 | 0.189 | 0.290 |
| *Porphyromonas sp. HOT 277* | 0.0 | 63.6 | <0.001 | 0.0000 | 0.3180 | 0.006 |  | 72.7 | 54.5 | 0.045 | 0.018 |
| *Porphyromonas sp. HOT 279* | 31.8 | 100.0 | <0.001 | 0.0210 | 0.8511 | <0.001 |  | 100.0 | 100.0 | 0.840 | 0.863 |
| *Prevotella buccalis* | 4.5 | 0.0 |  | 0.0003 | 0.0000 |  |  | 0.0 | 0.0 | 0.000 | 0.000 |
| *Prevotella denticola* | 0.0 | 9.1 |  | 0.0000 | 0.0968 |  |  | 18.2 | 0.0 | 0.194 | 0.000 |
| *Prevotella histicola* | 31.8 | 40.9 |  | 0.0057 | 0.0184 |  |  | 54.5 | 27.3 | 0.020 | 0.017 |
| *Prevotella intermedia* | 0.0 | 9.1 |  | 0.0000 | 0.0016 |  |  | 9.1 | 9.1 | 0.002 | 0.002 |
| *Prevotella maculosa* | 0.0 | 36.4 | 0.002 | 0.0000 | 0.0260 |  |  | 36.4 | 36.4 | 0.041 | 0.011 |
| *Prevotella melaninogenica* | 81.8 | 90.9 |  | 0.1747 | 0.2788 |  |  | 90.9 | 90.9 | 0.157 | 0.401 |
| *Prevotella micans* | 0.0 | 13.6 |  | 0.0000 | 0.0119 |  |  | 18.2 | 9.1 | 0.023 | 0.001 |
| *Prevotella oris* | 0.0 | 18.2 | 0.036 | 0.0000 | 0.0551 |  |  | 18.2 | 18.2 | 0.085 | 0.026 |
| *Prevotella oulorum* | 9.1 | 40.9 | 0.015 | 0.0004 | 0.0796 |  |  | 36.4 | 45.5 | 0.144 | 0.015 |
| *Prevotella pallens sp. HOT 310* | 4.5 | 18.2 |  | 0.0006 | 0.0163 |  |  | 18.2 | 18.2 | 0.004 | 0.030 |
| *Prevotella pleuritidis [NV]* | 0.0 | 9.1 |  | 0.0000 | 0.0008 |  |  | 9.1 | 9.1 | 0.001 | 0.01 |
| *Prevotella salivae* | 9.1 | 31.8 |  | 0.0035 | 0.0080 |  |  | 18.2 | 45.5 | 0.002 | 0.014 |
| *Prevotella scopos* | 0.0 | 13.5 |  | 0.0000 | 0.0031 |  |  | 18.2 | 9.1 | 0.003 | 0.003 |
| *Prevotella shahii* | 0.0 | 13.6 |  | 0.0000 | 0.0021 |  |  | 9.1 | 18.2 | 0.001 | 0.003 |
| *Prevotella sp. HOT 309* | 4.5 | 0.0 |  | 0.0050 | 0.0000 |  |  | 0.0 | 0.0 | 0.000 | 0.000 |
| *Prevotella sp. HOT 313* | 27.3 | 40.9 |  | 0.0352 | 0.0079 |  |  | 45.5 | 36.4 | 0.011 | 0.005 |
| *Prevotella sp. HOT 314* | 13.6 | 27.3 |  | 0.0013 | 0.0377 |  |  | 27.3 | 27.3 | 0.070 | 0.005 |
| *Prevotella sp. HOT 317* | 4.5 | 77.3 | <0.001 | 0.0001 | 0.2573 | <0.001 |  | 72.7 | 81.8 | 0.290 | 0.224 |
| *Propionibacterium propionicum* | 0.0 | 13.6 |  | 0.0000 | 0.0009 |  |  | 0.0 | 27.3 | 0.000 | 0.002 |
| *Ralstonia pickettii* | 9.1 | 9.1 |  | 0.0004 | 0.0011 |  |  | 18.2 | 0.0 | 0.002 | 0.000 |
| *Rothia aeria* | 40.9 | 95.5 | <0.001 | 0.0099 | 1.0099 | <0.001 |  | 90.9 | 100.0 | 1.002 | 1.017 |
| *Sanguibacter keddieii* | 9.1 | 0.0 |  | 0.0005 | 0.0000 |  |  | 0.0 | 0.0 | 0.000 | 0.000 |
| *Scardovia inopinata* | 0.0 | 4.5 |  | 0.0000 | 0.0012 |  |  | 0.0 | 9.1 | 0.000 | 0.002 |
| *Scardovia wiggsiae* | 4.5 | 9.1 |  | 0.0084 | 0.0022 |  |  | 9.1 | 9.1 | 0.003 | 0.002 |
| *Selenomonas artemidis* | 0.0 | 40.9 | 0.001 | 0.0000 | 0.0993 |  |  | 45.5 | 36.4 | 0.134 | 0.065 |
| *Selenomonas dianae* | 0.0 | 9.1 |  | 0.0000 | 0.0012 |  |  | 9.1 | 9.1 | 0.001 | 0.002 |
| *Selenomonas infelix sp. HOT 481_479* | 4.5 | 54.5 | <0.001 | 0.0003 | 0.0490 |  |  | 54.5 | 54.5 | 0.077 | 0.021 |
| *Selenomonas noxia* | 0.0 | 31.8 | 0.004 | 0.0000 | 0.1763 |  |  | 36.4 | 27.3 | 0.209 | 0.144 |
| *Selenomonas sp. HOT 126* | 0.0 | 36.4 | 0.002 | 0.0000 | 0.1075 |  |  | 36.4 | 36.4 | 0.195 | 0.020 |
| *Selenomonas sp. HOT 133* | 0.0 | 4.5 |  | 0.0000 | 0.0082 |  |  | 9.1 | 0.0 | 0.016 | 0.144 |
| *Selenomonas sp. HOT 136* | 0.0 | 18.2 | 0.036 | 0.0000 | 0.0011 | 0.038 |  | 9.1 | 27.3 | 0.001 | 0.002 |
| *Selenomonas sp. HOT 137* | 4.5 | 50.0 | 0.001 | 0.0002 | 0.0403 | 0.024 |  | 54.5 | 45.5 | 0.058 | 0.023 |
| *Selenomonas sp. HOT 140* | 0.0 | 36.4 | 0.002 | 0.0000 | 0.0186 | 0.036 |  | 45.5 | 27.3 | 0.017 | 0.021 |
| *Selenomonas sp. HOT 149* | 4.5 | 40.9 | 0.004 | 0.0006 | 0.0500 |  |  | 27.3 | 54.5 | 0.088 | 0.116 |
| *Selenomonas sp. HOT 478* | 0.0 | 4.5 |  | 0.0000 | 0.0007 |  |  | 0.0 | 9.1 | 0.000 | 0.001 |
| *Selenomonas sp. HOT 892* | 0.0 | 50.0 | <0.001 | 0.0000 | 0.0501 | 0.032 |  | 45.5 | 54.5 | 0.057 | 0.043 |
| *Selenomonas sp. HOT 143 / S. sputigena* | 9.1 | 40.9 | 0.015 | 0.0004 | 0.1213 |  |  | 45.5 | 36.4 | 0.207 | 0.034 |
| *SR1 [G-1] sp. HOT 345* | 0.0 | 18.2 | 0.036 | 0.0052 | 0.0000 |  |  | 27.3 | 9.1 | 0.006 | 0.005 |
| *SR1 [G-1] sp. HOT 875* | 0.0 | 18.2 | 0.036 | 0.0000 | 0.0047 |  |  | 18.2 | 18.2 | 0.003 | 0.006 |
| *Staphylococcus aureus* | 9.1 | 0.0 |  | 0.0034 | 0.0000 |  |  | 0.0 | 0.0 | 0.000 | 0.000 |
| *Staphylococcus epidermidis* | 63.6 | 4.5 | <0.001 | 0.0529 | 0.0004 |  |  | 9.1 | 0.0 | 0.001 | 0.000 |
| *Staphylococcus warneri* | 0.0 | 9.1 |  | 0.0000 | 0.0045 |  |  | 9.1 | 9.1 | 0.007 | 0.002 |
| *Stenotrophomonas maltophilia* | 18.2 | 0.0 |  | 0.0011 | 0.0000 |  |  | 0.0 | 0.0 | 0.000 | 0.000 |
| *Streptococcus anginosus* | 4.5 | 13.6 |  | 0.0051 | 0.0384 |  |  | 18.2 | 9.1 | 0.068 | 0.009 |
| *Streptococcus australis* | 54.5 | 90.9 | 0.007 | 0.1034 | 0.1439 |  |  | 90.9 | 90.9 | 0.079 | 0.209 |
| *Streptococcus mutans* | 0.0 | 81.8 | <0.001 | 0.0000 | 0.2216 |  |  | 72.7 | 90.9 | 0.036 | 0.407 |
| *S oligofermentans/S. cristatus/ S. australis* | 86.4 | 100.0 |  | 0.4869 | 2.3261 | <0.001 |  | 100.0 | 100.0 | 2.343 | 2.309 |
| *S oralis/S. mitis/S. mitis bv2/S. infantis* | 100.0 | 100.0 |  | 62.0337 | 38.3072 | <0.001 |  | 100.0 | 100.0 | 35.238 | 41.376 |
| *S. parasanguinis I/Streptococcus sp. HOT 66* | 36.4 | 90.9 | <0.001 | 0.4873 | 0.2293 |  |  | 90.9 | 90.9 | 0.280 | 0.176 |
| *Streptococcus parasanguinis II* | 59.1 | 77.3 |  | 1.0672 | 0.0830 |  |  | 81.8 | 72.7 | 0.072 | 0.094 |
| *S. peroris/Streptococcus sp. HOT 68* | 100.0 | 100.0 |  | 11.3865 | 0.2709 | <0.001 |  | 100.0 | 100.0 | 0.266 | 0.276 |
| *Streptococcus pyogenes* | 0.0 | 4.5 |  | 0.0000 | 0.0006 |  |  | 0.0 | 9.1 | 0.000 | 0.001 |
| *Streptococcus sanguinis* | 100.0 | 100.0 |  | 0.1053 | 1.5617 | <0.001 |  | 100.0 | 100.0 | 2.039 | 1.085 |
| *Streptococcus sobrinus* | 4.5 | 13.6 |  | 0.0003 | 0.2187 |  |  | 9.1 | 18.2 | 0.435 | 0.003 |
| *Streptococcus sp. HOT 057* | 31.8 | 31.8 |  | 0.0207 | 0.0095 |  |  | 36.4 | 27.3 | 0.005 | 0.014 |
| *Streptococcus sp. HOT 058* | 95.5 | 68.2 | 0.019 | 0.0500 | 0.0125 |  |  | 54.5 | 81.8 | 0.010 | 0.014 |
| *Streptococcus sp. HOT 071* | 100.0 | 81.8 | 0.036 | 0.0631 | 0.0269 | <0.001 |  | 81.8 | 81.8 | 0.020 | 0.034 |
| *Streptococcus sp. HOT 074* | 100.0 | 100.0 |  | 1.8198 | 0.1961 | 0.006 |  | 100.0 | 100.0 | 0.48 | 0.145 |
| *Streptococcus sp. HOT 431* | 95.5 | 95.5 |  | 0.0243 | 0.0039 | 0.033 |  | 90.9 | 100.0 | 0.015 | 0.034 |
| *TM7 [G-1] sp. HOT 346* | 4.5 | 13.6 |  | 0.0001 | 0.0039 |  |  | 9.1 | 18.2 | 0.001 | 0.003 |
| *TM7 [G-1] sp. HOT 347* | 0.0 | 22.7 | 0.018 | 0.0000 | 0.0060 |  |  | 9.1 | 36.4 | 0.007 | 0.005 |
| *TM7 [G-1] sp. HOT 348* | 0.0 | 40.9 | 0.001 | 0.0000 | 0.0115 | 0.030 |  | 54.5 | 27.3 | 0.016 | 0.007 |
| *TM7 [G-1] sp. HOT 352* | 4.5 | 13.6 |  | 0.0017 | 0.0018 |  |  | 9.1 | 18.2 | 0.001 | 0.002 |
| *TM7 [G-1] sp. HOT 353* | 0.0 | 13.6 |  | 0.0000 | 0.0027 |  |  | 18.2 | 9.1 | 0.002 | 0.004 |
| *TM7 [G-3] sp. HOT 351* | 4.5 | 50.0 | 0.001 | 0.0070 | 0.0387 |  |  | 54.5 | 45.5 | 0.036 | 0.041 |
| *Veillonella atypica* | 50.0 | 27.3 |  | 0.0387 | 0.0396 |  |  | 27.3 | 27.3 | 0.007 | 0.005 |
| *Veillonella denticariosi* | 0.0 | 22.7 | 0.018 | 0.0000 | 0.0059 |  |  | 9.1 | 36.4 | 0.005 | 0.001 |
| *Veillonella dispar* | 81.8 | 100.0 | 0.036 | 3.8187 | 0.4093 |  |  | 100.0 | 100.0 | 0.011 | 0.288 |
| *Veillonella parvula* | 18.2 | 100.0 | <0.001 | 0.0011 | 0.6804 | <0.001 |  | 100.0 | 100.0 | 0.948 | 0.413 |
| *Veillonella rogosae* | 18.2 | 63.6 | 0.002 | 0.1376 | 0.2108 |  |  | 63.6 | 63.6 | 0.151 | 0.270 |
| *Veillonella sp. HOT 780* | 90.9 | 100.0 |  | 2.6230 | 1.1344 |  |  | 100.0 | 100.0 | 1.142 | 1.127 |
| *Veillonella sp. HOT 917* | 9.1 | 4.5 |  | 0.0068 | 0.0013 |  |  | 9.1 | 0.0 | 0.003 | 0.000 |
