# Supplementary material for: Maturation of Oral Microbiota in Children with or without Dental Caries
Source: PLoS One. 2015 May 28;10(5):e0128534. doi: 10.1371/journal.pone.0128534 (PMC4447273; doi:10.1371/journal.pone.0128534)
Supplement: S3 Table — The oral microbiota was determined by (A) 454 FLX+ pyrosequencing and (B) the HOMIM microarray in samples from 3-months old infants and repeated when the child was 3-years of age. Core microbiota refers to bacteria detected in all children. Age characteristic species/phylotypes from (A) pyrosequencing and (B) HOMIM analyses were identified by PLS modeling with species/phylotypes as the independent block and age as the dependent variable. Species/phylotypes where the 95% CI did not include zero, i.e. statistically significant, are listed in alphabetical order. (DOCX) [file pone.0128534.s003.docx]

**S3 Table. Core microbiota and age associated species/phylotypes in 3-months and 3-years old children, respectively, in the MamBa study cohort by (A) pyrosequencing and (B) the HOMIM microarray.**

| **A. Pyrosequencing detected species/phylotypes** | | | | |
| --- | --- | --- | --- | --- |
| **Core microbiota by age group** | |  | **Age group characteristics by PLS** | |
| **3 months of age** | **3 years of age** |  | **3 months of age** | **3 years of age** |
|  | *Abiotrophia defectiva* |  | *Streptococcus peroris* | *Abiotrophia defective* |
|  | *Actinomyces johnsonii sp. HOT 171_849* |  | *S.oralis/S.mitis/S.mitis bv2/S.infantis* | *Actinobaculum sp. HOT 183* |
|  | *Actinomyces oris* |  | *Streptococcus sp. HOT 071* | *Actinomyces johnsonii sp. HOT 171_849* |
|  | *Actinomyces sp. HOT 180* |  | *Streptococcus sp. HOT 074* | *Actinomyces oris* |
|  | *Bergeyella sp. HOT 322* |  | *Streptococcus sp. HOT 431* | *Actinomyces sp. HOT 171* |
|  | *Capnocytophaga sp. HOT 864* |  | *Lactobacillus crispatus* | *Actinomyces sp. HOT 175* |
|  | *Capnocytophaga sputigena* |  | *Actinomyces sp. HOT 181* | *Actinomyces sp. HOT 177* |
|  | *Corynebacterium durum* |  | *Veillonella sp. HOT 780* | *Actinomyces sp. HOT 178* |
|  | *Fusobacterium nucleatum ss polymorphum* |  |  | *Actinomyces sp. HOT 877* |
| *Gemella haemolysans* | *Gemella haemolysans* |  |  | *Aggregatibacter paraphrophilus* |
| *Haemophilus influenzae* | *Haemophilus influenzae* |  |  | *Aggregatibacter segnis* |
|  | *Haemophilus parainfluenzae* |  |  | *Aggregatibacter sp. HOT 458* |
|  | *Haemophilus sp. HOT 036* |  |  | *Aggregatibacter sp. HOT 898* |
|  | *Kingella oralis* |  |  | *Alloprevotella sp. HOT 308* |
|  | *Lautropia mirabilis* |  |  | *Bergeyella sp. HOT 322* |
|  | *Neisseria elongata* |  |  | *Bergeyella sp. HOT 907* |
|  | *Neisseria flavescens* |  |  | *Campylobacter concisus* |
|  | *Neisseria mucosa_flava* |  |  | *Campylobacter rectus* |
|  | *Porphyromonas sp. HOT 279* |  |  | *Campylobacter showae_rectus* |
|  | *S oligofermentans/S. cristatus/ S. australis* |  |  | *Capnocytophaga granulosa* |
| *S. oralis/S. mitis/S. mitis bv2/S. infantis* | *S. oralis/S. mitis/S. mitis bv2/S. infantis* |  |  | *Capnocytophaga leadbetteri* |
|  | *S. peroris/Streptocococcus sp. HOT 68* |  |  | *Capnocytophaga sp. HOT 326* |
| *Streptococcus sanguinis* | *Streptococcus sanguinis* |  |  | *Capnocytophaga sp. HOT 332* |
| *Streptococcus sp. HOT 074* | *Streptococcus sp. HOT 074* |  |  | *Capnocytophaga sp. HOT 864* |
| *Streptococcus sp. HOT 071* | *Veillonella dispar* |  |  | *Capnocytophaga sp. HOT 902_336* |
|  | *Veillonella parvula* |  |  | *Capnocytophaga sp. HPT 903_412* |
|  | *Veillonella sp. HOT 780* |  |  | *Capnocytophaga sputigena* |
|  |  |  |  | *Clostridiales [F-2][G-1] sp. HOT 075* |
|  |  |  |  | *Corynebacterium durum* |
|  |  |  |  | *Dialister invisus* |
|  |  |  |  | *Eubacterium [XIVa][G-1] saburreum* |
|  |  |  |  | *Fusobacterium naviforme* |
|  |  |  |  | *Fusobacterium nucleatum ss polymorphum* |
|  |  |  |  | *Fusobacterium nucleatum ss vincentii* |
|  |  |  |  | *Gemella morbillorum* |
|  |  |  |  | *Granulicatella adiacens [para-adiacens]* |
|  |  |  |  | *Granulicatella elegans* |
|  |  |  |  | *Haemophilus haemolyticus* |
|  |  |  |  | *Haemophilus influenzae* |
|  |  |  |  | *Haemophilus parainfluenzae* |
|  |  |  |  | *Haemophilus sp. HOT 035* |
|  |  |  |  | *Haemophilus sp. HOT 908* |
|  |  |  |  | *Kingella denitrificans* |
|  |  |  |  | *Kingella oralis* |
|  |  |  |  | *Lachnospiraceae [G-2] sp. HOT 088* |
|  |  |  |  | *Lautropia mirabilis* |
|  |  |  |  | *Leptotrichia goodfellowii* |
|  |  |  |  | *Leptotrichia hofstadii sp. HOT 909* |
|  |  |  |  | *Leptotrichia shahii* |
|  |  |  |  | *Leptotrichia sp. HOT 219* |
|  |  |  |  | *Leptotrichia sp. HOT 392* |
|  |  |  |  | *Leptotrichia sp. HOT 879* |
|  |  |  |  | *Leptotrichia sp. HOT 212_217* |
|  |  |  |  | *Leptotrichia sp. HOT 225 / L. buccalis* |
|  |  |  |  | *Neisseria elongata* |
|  |  |  |  | *Neisseria mucosa / N. flava* |
|  |  |  |  | *Neisseria subflava* |
|  |  |  |  | *Porphyromonas catoniae* |
|  |  |  |  | *Porphyromonas sp. HOT 277* |
|  |  |  |  | *Porphyromonas sp. HOT 279* |
|  |  |  |  | *Prevotella maculosa* |
|  |  |  |  | *Prevotella sp. HOT 317* |
|  |  |  |  | *Propionibacterium propionicum* |
|  |  |  |  | *Rothia aeria* |
|  |  |  |  | *S oligofermentans_cristatus_australis* |
|  |  |  |  | *Selenomonas sp. HOT 137* |
|  |  |  |  | *Selenomonas sp. HOT 892* |
|  |  |  |  | *Selenomonas sputigena sp. HOT 143* |
|  |  |  |  | *SR1 [G-1] sp. HOT 345* |
|  |  |  |  | *Streptococcus mutans* |
|  |  |  |  | *Streptococcus sanguinis* |
|  |  |  |  | *TM7 [G-1] sp. HOT 348* |
|  |  |  |  | *TM7 [G-3] sp. HOT 351* |
|  |  |  |  | *Veillonella parvula* |

| **B. HOMIM microarray** | |
| --- | --- |
| **3 months of age** | **3 years of age** |
| *Burkholderia sp. HOT 406* | *Abiotrophia defectiva HOT 389* |
| *Gemella sanguinis HOT 757* | *Actinomyces meyeri and odontolyticus HOT 671_701* |
| *Campylobacter concisus and rectus HOT 575_748* | *Actinomyces naeslundii HOT 176* |
| *Fusobacterium naviforme and nucleatum ss vincentii HOT 200* | *Bergeyella sp. HOT 322* |
| *Streptococcus cristatus and sp. HOT 058_578* | *Campylobacter Cluster II HOT 580_748_763* |
| *Capnocytophaga ochracea and sp. HOT 323_ 326_700* | *Campylobacter concisus HOT 575* |
| *Aggregatibacter actinomycetemcomitans HOT 531* | *Campylobacter gracilis HOT 623* |
| *Propionibacterium propionicum HOT 739* | *Campylobacter showae HOT 763* |
| *Rothia mucilaginosa HOT 681* | *Capnocytophaga sputigena HOT 775* |
| *Actinomyces Cluster II HOT 180_ 181_671_701* | *Cardiobacterium hominis HOT 633* |
| *Cardiobacterium valvulum HOT 540* | *Eikenella corrodens and Kingella denitrificans and sp HOT 012_577_582* |
| *Streptococcus pyogenes HOT 745* | *Fusobacterium periodontium HOT 201* |
| *Filifactor alocis HOT 539* | *Gemella morbillorum HOT 046* |
| *Streptococcus downei HOT 594* | *Granulicatella adiacens HOT 534* |
| *Porphyromonas endodontalis and sp. HOT 273_ 285_395* | *Granulicatella adiacens and elegans HOT 534_596* |
| *Actinomyces sp. HOT 177* | *Granulicatella elegans HOT 596* |
| *Campylobacter gracilis HOT 623* | *Haemophilus parainfluenzae HOT 718* |
| *Megasphaera sp. HOT 123* | *Haemophilus sp. HOT 036* |
| *Prevotella Cluster III HOT 306_31_ 313* | *Kingella denitrificans HOT 582* |
| *SR1[G-1] sp. HOT 345* | *Lachnospiraceae[G-3] sp. HOT 100* |
| *Streptococcus infantis and sp. HOT 065_638* | *Lautropia mirabilis HOT 022* |
| *Tannerella forsythensis HOT 613* | *Leptotrichia hofstadii and sp. HOT 223_224* |
| *Streptococcus parasanguis I and II HOT 057_411_721* | *Neisseria Cluster II HOT 014_609_682_764* |
| *Slackia exigua HOT 602* | *Neisseria elongata HOT 598* |
|  | *Neisseria flavescens HOT 610* |
|  | *Porphyromonas catoniae and sp. HOT 279_283* |
|  | *Prevotella melaninogenica and sp.HOT 298_469* |
|  | *Rothia dentocariosa and mucilaginosa HOT 587_681* |
|  | *Streptococcus anginosus and gordonii HOT 543_622* |
|  | *Streptococcus constellatus and intermedius HOT 576_644* |
|  | *Streptococcus cristatus and sp. HOT 058_578* |
|  | *Streptococcus mitis bv2 and sp. HOT 069_398* |
|  | *Streptococcus oralis and sp. HOT 064_707* |
|  | *Streptococcus salivarius and sp. HOT 067_755* |
|  | *Treponema denticola HOT 584* |
